# Feeding ecology of scolopendromorphs: integrating a global literature review with Japanese citizen-sourced data

**DOI:** 10.7717/peerj.20482

**Published:** 2026-01-05

**Authors:** Ryosuke Uno

**Affiliations:** Zoology, Kyoto University, Kyoto, Japan

**Keywords:** Centipede ecology, Citizen science, Feeding interactions, Food web, Eltonian shortfall, Scolopendromorpha, Social media mining, Literature survey

## Abstract

Knowledge of food habits is essential for ecological research, yet those are often assessed based on general assumptions rather than evidence, especially in secretive animals that are difficult to observe in the wild, such as centipedes. This leads to underestimation and mischaracterisation of their ecological roles. To address this problem, the present study evaluates the trophic interactions of scolopendromorphs by a dual approach, which integrates a global literature review with analysis of Japanese citizen-sourced data to examine the diets of scolopendromorphs. A total of 76 publications were systematically reviewed, and 102 reported predation events were analysed to assess publication trends and predator–prey size ratios. Concurrently, 8,684 entries from social media and citizen science platforms were mined, yielding 1,130 predation records that were used to construct a detailed prey inventory for Japanese scolopendromorphs. The literature review revealed that scolopendromorphs consume small prey when targeting mammals and amphibians but are capable of preying on large reptiles comparable to themselves, at least regarding body length. Despite the increase in recent publications, it is highly probable that available reports are biased towards vertebrate prey. In contrast, although the citizen-sourced data lacked quantitative metrics such as body size, they encompassed a much broader array of trophic interactions, including the consumption of animals, both alive and dead, and plant material, many of which are not covered in the literature. The citizen-sourced data revealed ecologically notable interactions including cross predation, foraging on spider webs, and ingestion of venomous animals. Collectively, these findings highlight the remarkably broad foraging versatility of scolopendromorphs and ascertain a potential risk of drawing ecological inferences from unverified assumptions or a biased subset of evidence. By integrating a traditional literature review with analysis of citizen-sourced data, the present study not only provides a more comprehensive portrayal of the feeding ecology of scolopendromorphs but also illustrates a promising methodology for uncovering the structure of food webs for secretive animals, for which observations depend on chance encounters.

## Introduction

Food habits represent one of the most fundamental aspects of biotic interactions. Food habits govern nutritional hierarchies and underpin our understanding of diverse ecological processes, including population and food web dynamics, and the evolution of behavioural, morphological, and physiological adaptations in both predators and prey (Polis & Strong, 1996; Thompson et al., 2007). The most reliable data on food habits are obtained through direct observations in natural environments, free from the biases of laboratory conditions (Sih, 1980; Nielsen et al., 2018; Nordberg, Edwards & Schwarzkopf, 2018). However, the spatiotemporal unpredictability of predation events makes systematic study extremely challenging (Barve, 2014; Nordberg, Edwards & Schwarzkopf, 2018; Maritz & Maritz, 2020; Díaz et al., 2024). Consequently, food habits of many animals, even well-known species, are often assessed based on general assumptions without evidence, especially when they have secretive habits. Lack of knowledge about biotic interactions, such as trophic relationships, represents a deeper, more complex, and important gap in our understanding of biodiversity—known as the Eltonian shortfall (Hortal et al., 2015)—which could mislead the subsequent related fields, with potentially serious consequences (Tylianakis et al., 2008; Fleming & Bateman, 2016).

Centipedes (Myriapoda: Chilopoda) are a group of venomous arthropods well-known to both biologists and non-biologists (Shelley, 2002). In particular, the order Scolopendromorpha, which includes many of the largest centipede species, is found on every continent except for Antarctica (Bonato & Zapparoli, 2011). It is the most familiar type of centipede and is notorious for its extremely painful bites (Veraldi, Çuka & Gaiani, 2014). The order comprises five families: Cryptopidae, Mimopidae, Plutoniumidae, Scolopendridae, and Scolopocryptopidae. Fewer than ten species are present both in Mimopidae and Plutoniumidae (Bonato et al., 2016). Cryptopidae and Scolopocryptopidae include approximately 170 and 80 species, respectively (Edgecombe & Bonato, 2011). Species in these four families either lack eyes or have reduced eyes and are thought to be primarily adapted to life within the humus layer and soil (Lewis, 1981). In contrast, Scolopendridae, with approximately 400 species, displays substantial morphological and ecological diversity, including variation in size and colouration and adaptation to semi-aquatic habitats (Lewis, 1981; Edgecombe & Bonato, 2011; Siriwut et al., 2016; Tsukamoto et al., 2021). All species of these five families have traditionally been regarded as opportunistic predators, feeding on insects and earthworms on the forest floor (Lewis, 1981); however, an increasing number of recent reports have documented instances of vertebrate predation (Valdez, 2020). Given that invertebrates are generally positioned as prey of vertebrates in terrestrial food webs (McCormick & Polis, 1982; Noronha et al., 2015), such inversions of trophic hierarchy by scolopendromorphs have attracted considerable attention, particularly in terms of their frequency and the complexity of their impact on local food webs (Jestrzemski & Schütz, 2016; Nordberg, Edwards & Schwarzkopf, 2018; Valdez, 2020; Deimezis-Tsikoutas, Kapsalas & Pafilis, 2020; Halpin et al., 2021; Hodges & Goodyear, 2021). However, most reports on interactions between scolopendromorphs and vertebrates have been published sporadically in journals focused on vertebrates and there is a growing need for a systematic review to organise and synthesise this scattered body of information.

In order to contextualise this need, it is important to consider the methodological approaches that have been employed in centipede feeding studies. Food habits are typically investigated through direct observations, feeding mark surveys, alimentary canal content or faecal analysis, stable isotope ratio analysis, and prey DNA detection. To my knowledge, feeding mark surveys and faecal analysis have not been applied to centipedes. Analysis of alimentary canal contents is difficult for prey identification due to the gnawing feeding style of centipedes (Balbiani, 1890; Lewis, 1966; Plateau, 1878). DNA barcoding can provide highly accurate species identification based on molecular data; however, it cannot distinguish secondary predation (*i.e*., consuming food items eaten by the prey animal) from actual hunting (Deagle et al., 2018; Sheppard & Harwood, 2005; Tercel, Symondson & Cuff, 2021). Stable isotope ratio analysis is highly effective for determining trophic levels of target animals within an ecosystem; however, it is not suitable for resolving interspecies interactions (Ihara, 2014). Ultimately, the effective application of these methods requires a prior qualitative understanding of centipede prey through direct field observations, as demonstrated by Tercel et al. (2024) and Halpin et al. (2021). Thus, although studies on food habits of scolopendromorphs have begun to involve molecular and isotope analyses, a comprehensive understanding of prey types, taxonomic groups of prey, and regional characteristics based on qualitative data is still greatly needed to establish a solid foundation for quantitative analyses.

Such foundational knowledge of centipede prey diversity and feeding interactions is particularly relevant in light of recent concerns about invasive centipede species. Although the vast majority of scolopendromorphs are not invasive, some species exhibit highly invasive ecology and have been increasingly recognised to cause severe impacts on ecosystems into which they have been introduced (Stoev et al., 2010; Prötzel, Randriamanana & Glaw, 2023). For example, on Christmas Island, an introduced centipede, *Scolopendra subspinipes*, has been reported as a possible driver of the extinction of the endemic blue-tailed skink (*Cryptoblepharus egeriae*) (Emery et al., 2021a, 2021b). Considering these backgrounds, addressing the Eltonian shortfall in scolopendromorphs and elucidating their feeding ecology is critical for deepening our understanding of biodiversity and addressing the ongoing biodiversity crisis in human-modified ecosystems.

Citizen science is widely recognised as one of the most powerful tools for collecting data on geographic distributions and abundance of organisms, *i.e*., reducing the Wallacean and Prestonian shortfalls, respectively (Dickinson, Zuckerberg & Bonter, 2010; Hortal et al., 2015; Pocock et al., 2018; Groom et al., 2021; Edwards et al., 2021; Fraisl et al., 2022). In recent years, mining of photographic data accumulated on citizen science platforms and social media has uncovered previously undocumented ecological interactions in birds, reptiles, amphibians, and rove beetles (Maritz & Maritz, 2020; Díaz et al., 2024; Pernat et al., 2024a, 2024b; Panter et al., 2024; Hu, Hsiao & Solodovnikov, 2025). These studies demonstrate that citizen science can also serve as an effective means of addressing the Eltonian shortfall. On the other hand, citizen science has been frequently criticised because of its potential limitations in data accuracy and concerns regarding open access, given that the information is typically gathered by non-experts (Guerrini et al., 2018; Adler, Green & Şekercioğlu, 2020; Edwards et al., 2021). Moreover, when mining photographs in citizen science platforms and social media, data on species, time, date, and location are not collected in a standardised format, unlike in organised citizen science projects. As a result, the effective extraction and selection of information aligned with specific research objectives requires robust filtering methods, including manual screening (Naude et al., 2019; Panter et al., 2024) and artificial intelligence approaches such as image recognition (Edwards et al., 2021; Pernat et al., 2024a).

In recent years, citizen science has increasingly been utilised to address the Wallacean and Prestonian shortfalls in myriapods, including scolopendromorphs, particularly in relation to rare species (Bonato et al., 2017; Wesener, 2018; Neu et al., 2022; So et al., 2022; Faraone et al., 2024). However, studies employing citizen science to investigate biotic interactions in myriapods remain scarce. Consequently, the effectiveness and applicability of citizen science in addressing the Eltonian shortfall in this group have yet to be examined. Despite their predominantly nocturnal and secretive habits, many scolopendromorphs are relatively familiar to the general public, and their predatory behaviour is readily observable, as they restrain their prey using their forelegs (Elzinga, 1994; Manton, 1997). These characteristics make them particularly well suited as model organisms for detecting trophic interactions through visual media. By leveraging these unique characteristics of scolopendromorphs, the present study expects to serve as a model case for citizen science in uncovering biotic interactions of elusive predators.

The aims of this study are to assess the current knowledge of the food habits of scolopendromorphs by providing a review of global literature and a detailed prey list of Japanese species derived from citizen-sourced data, and to establish a baseline that facilitates future quantitative studies of their feeding ecology. The geographical restriction of the citizen science survey to Japan is based on two reasons. Firstly, in Japan, the taxonomic frameworks for scolopendromorphs and their potential prey, such as insects, reptiles, and amphibians, are relatively well established (The Editorial Committee of Catalogue of the Insects of Japan, 2013; Shinohara, Takano & Ishii, 2015; Herpetological Society of Japan, 2021; Union of Japanese Societies for Systematic Biology, 2025). Consequently, species-level analysis can be conducted with a low risk of misidentification. Secondly, substantial knowledge has already been accumulated regarding the predators of Japanese centipedes, including various species of amphibians, birds, and mammals (Uno, 2024). Therefore, elucidating food habits of Japanese scolopendromorphs is expected to contribute significantly to the further advancement of community ecological research, by serving as a model for understanding nutrient flows from soil ecosystems to terrestrial ecosystems. The present study highlights the effectiveness of citizen science on secretive animals, in which observations depend heavily on chance encounters.

## Materials and Methods

### Definitions

In this study, the term ‘foraging’ refers to all events of centipedes eating food. The term ‘predation’ refers to foraging events on live animals and the following sequence is expected: capture-transport-consumption. Records of centipedes seizing or carrying an animal were also categorised as predation, although it is not known whether the centipedes actually ate the prey afterwards. Feeding on plant matter and human-derived items is not categorised as predation. Human-derived items comprise food discarded after harvesting or cooking for human consumption, as well as food intentionally thrown away into the natural environment by humans. Examples include hamburgers on the street, fish discarded by anglers, and bananas used in insect traps. The term ‘scavenging’ refers to foraging events on dead animals, in which the capture phase is lacking. If the prey was immobile when an observer found it, but the prey appeared fresh (*e.g*., dripping blood or being restrained by multiple legs of the centipede), it was categorised as predation. Because distinguishing between living and dead animals in visual media is challenging, the status of the prey (alive or dead) was determined by comprehensively combining its appearance (*e.g*., degree of desiccation, discolouration, body loss) with information provided in the observer’s text. For specific information on the circumstances and surroundings during the observation of predation events, refer to the respective sources.

### Literature survey of prey of scolopendromorphs

Literature on the order Scolopendromorpha was reviewed to pick up publications that focused on foraging events under natural conditions. Articles were compiled through queries of Web of Science, JSTOR, and Google Scholar for titles, abstracts, and full texts using the search terms ‘centipede’ or ‘Scolopendromorpha’ combined with ‘feeding’, ‘prey’, or ‘predation’, with no restrictions on year or language. The reference lists of all articles found in the above search were cross-checked. To examine temporal trends in publication frequency, the collected literature was categorised by publication year and analysed using the Mann–Kendall trend test.

Data collected from the literature were species name, prey and predator size, foraging environment, date and time, and study site. If centipede behaviour during foraging was reported, a brief description was included as notes. When body length of only either the prey or the centipede was recorded and an image featuring both animals was presented, the size of the other species was roughly estimated using the “Set Scale” function in ImageJ (Schneider, Rasband & Eliceiri, 2012). The scale was defined based on the known size of one animal to calculate the size per pixel. The size data were utilised to calculate predator–prey size ratio (PPSR) between the prey and centipedes. For prey taxa with a sufficiently large sample size (*n* > 10), comparisons of PPSR were made using the Wilcoxon rank-sum test, implemented in R (version 4.3.2; R Core Team, 2023). When recording the foraging environment, the phases that occurred there (capture, transport, or consumption) were also recorded.

Literature on food habits of scolopendromorphs based on the methods other than direct field observations (*e.g*., gut content analysis, DNA barcoding, and stable isotope analysis) is not included in this review, because the aim of the present study was to compile only the most direct and unambiguous evidence to establish a robust baseline dataset. These records have instead been compiled separately in the Supplemental Files for reference.

### Analysis of citizen-sourced data

Visual media featuring the class Chilopoda were collected to compile instances focusing on foraging events observed under natural conditions in Japan. Data mining was conducted across four major social media platforms with particularly high usage in Japan—Instagram (https://www.instagram.com), X (formerly Twitter) (https://x.com), Facebook (https://www.facebook.com), and YouTube (https://www.youtube.com)— as well as on websites and online stock photo sources *via* Google Images (https://images.google.com). In addition, raw photographic datasets were obtained from iNaturalist (https://www.inaturalist.org) and Japan-specific citizen science platforms, including Biome (https://biome.co.jp) and Ikimono Log (https://ikilog.biodic.go.jp). The citizen-sourced data were incorporated into the dataset (Uno, 2023) up to 31 July 2024. Terms and method used to search and collect visual media data followed Uno (2024), but only terms related to Chilopoda were employed.

Items collected for the visual media data included the source of the data, URL, username, presence or absence of video, species name, foraging environment, developmental stage, date and time, and the location of the observation. Dates and times were recorded if they could be determined from the accompanying text, but day–night distinction was made based on the ambient light condition in the visual media. Locations were recorded if they could be determined from the text or other available information. If particularly noteworthy foraging events were recorded, additional information regarding the circumstances was obtained by contacting the original contributors.

Identification of centipedes shown in visual media was conducted visually, and prey items were identified with occasional assistance from several experts. However, when the prey in the visual media was too small, was engulfed by a centipede, or was largely consumed, and when the media itself was unclear and poorly visible, we retained the taxon at a higher level of classification.

## Results

### Literature survey on diet of scolopendromorphs

**Figure 1:**
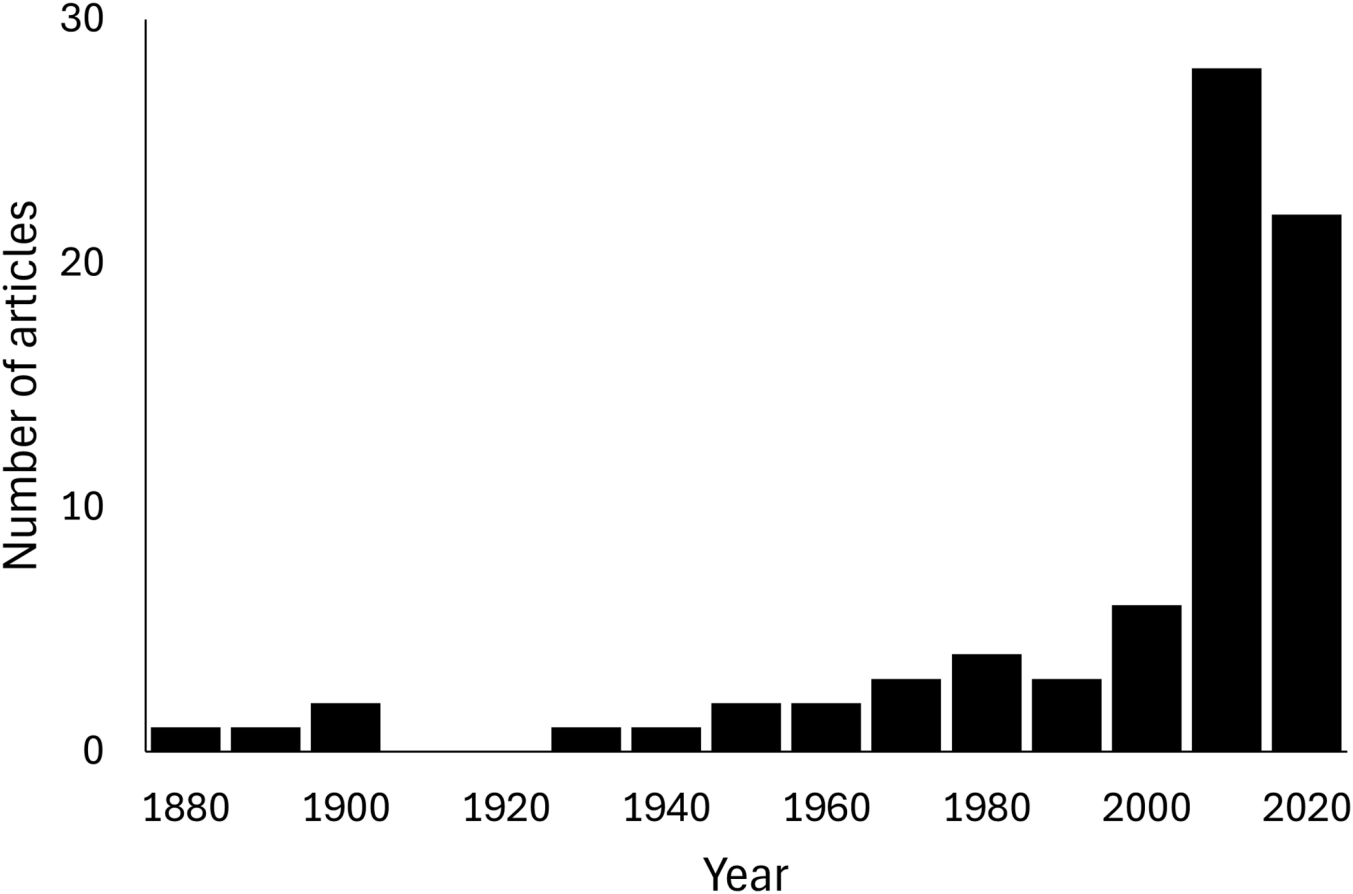
Yearly trend in the number of articles that report predation events of the order Scolopendromorpha under natural conditions. Mann–Kendall trend test, z = 6.3027, *p* < 0.0001, S = 2,696.

A total of 102 predation events of Scolopendromorpha were found in 76 articles (Supplement 1). No field observations of feeding on plant material or carcasses were confirmed from the literature. A significant upward trend over years was found in the number of articles reporting predation of scolopendromorphs under natural conditions (Mann-Kendall trend test, z = 6.3743, *p* < 0.001, S = 2,727, Fig. 1). All data from the literature review are publicly available to ensure reproducibility and transparency (DOI: https://doi.org/10.5281/zenodo.16021806).

The literature documented 79 prey species from 49 families for 25 species from four scolopendromorph families (Supplement 1). The most common predator was *S. subspinipes* occupying 9.80% of all documented predation events, followed by *S. heros* with 7.84%.

**Figure 2:**
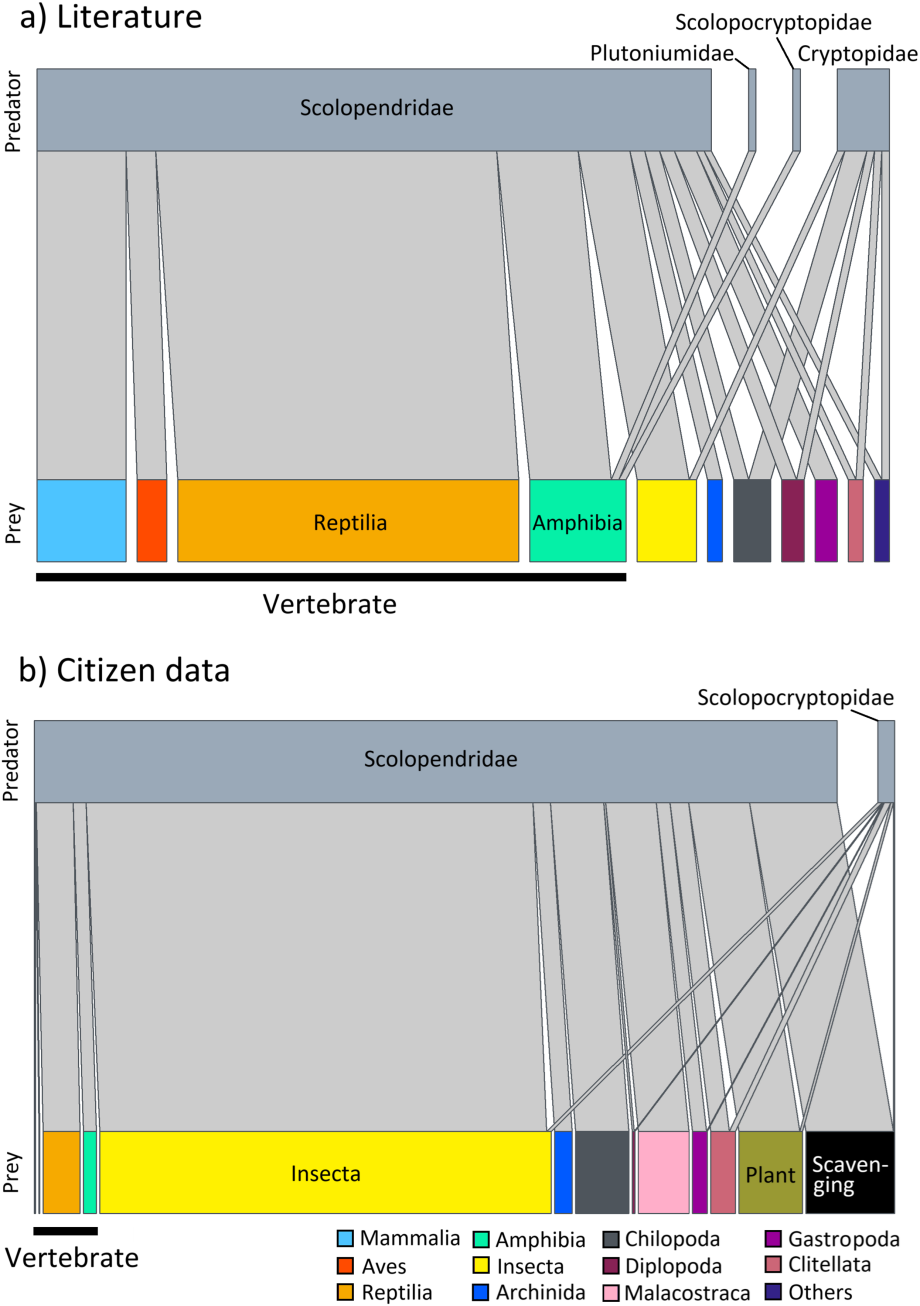
Interaction network between families of Scolopendromorpha (top bars) and classes of their prey (bottom bars). (A) based on global literature, and (B) based on citizen data in Japan.

**Figure 3:**
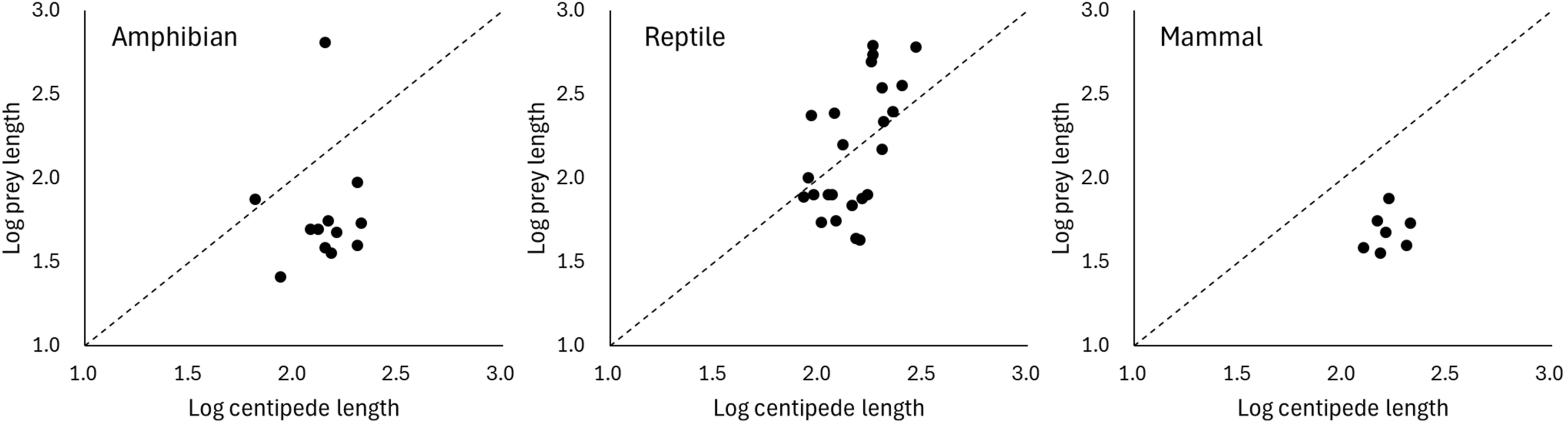
Relationships between the size of scolopendromorph predators and that of prey. The broken line represents where the size of predator is equal to that of prey.

Predation on vertebrate prey was confirmed in 77 events, whereas that on invertebrate prey was confirmed in 25 events, indicating a heavy bias in the literature towards documenting vertebrate predation (Fig. 2A). The most common class of prey, both in terms of number of reports and species, was reptiles with 47.06% of all documented predation events, followed by amphibians with 12.75%, and mammals with 11.76%. PPSR for these three prey taxa is shown in Fig. 3. Regarding the reptile prey consumed, PPSR was close to 1 in many instances, with no significant differences between their body size (Wilcoxon rank sum, W = 315.5, *p* = 0.578). Regarding the amphibian and mammalian prey consumed, PPSR with scolopendromorphs was mostly less than 1, with significant differences between their body size (Wilcoxon rank sum, amphibian: W = 129, *p* = 0.001; mammal: W = 49, *p* < 0.001).

Sixty-one vertebrate species from 32 families were obtained as prey animals (Supplement 1). The most common vertebrate prey taxon was reptiles, in which 52.08% of the observations were lizards and the remaining were snakes. Approximately three-quarters of amphibian prey were frogs (76.92%), and the majority of mammalian prey consisted of bats (83.33%). Of the predation events for which the date and time were recorded, 50.72% were observed during the day. Seven species (*Ethmostigmus rubripes*, *S. dehaani, S. gigantea*, *S. heros*, *S. mutilans*, *S. subspinipes* and *S. sumichrasti*) were observed foraging both during the day and at night. Although the foraging environments were diverse, ground (18.75%) was the most common during the capture phase, and tree trunk (11.29%) was the most common during the consumption phase.

Food items of scolopendromorphs obtained through methods other than direct field observation were identified in eight publications, comprising 58 instances in total. These records were obtained through three indirect methods: the analysis of gut contents, DNA barcoding, and stable isotopes. They are presented in Supplement 2.

### Diet of Japanese scolopendromorphs based on citizen data

The citizen science survey resulted in a database of 1,130 foraging events of the class Chilopoda from 8,496 images and 188 videos. These records comprised 188 photographs of Scutigeromorpha, 12 of Lithobiomorpha, 922 of Scolopendromorpha, and seven of Geophilomorpha. Among them, prey items of scolopendromorphs—the primary focus of this study—were identified as 118 species from 88 families, from which the prey list was compiled for the Japanese scolopendromorphs: *S. mutilans*, *S. japonica*, *S. morsitans*, *S. alcyona*, *S. subspinipes*, *Scolopendra* sp., *Otostigmus scaber*, and *Scolopocryptops* sp. (Supplement 1). Here, “sp.” refers to individuals identified only to the genus level and not to a specific species. All data from the citizen science survey are publicly available (DOI: https://doi.org/10.5281/zenodo.16021806).

Predation by Scolopendromorpha was confirmed in 696 events (75.49% of foraging events). The most common centipede predator was *S. mutilans* with 71.98% of predation events, followed by *S. japonica*. The most common class of prey, both in terms of number of reports and species, was insects, occupying 64.94% of all predation events, followed by centipedes with 8.33%, malacostracans with 7.90%, and reptiles with 5.75% (Table 1 and Fig. 2B).

**Table 1:** List of prey items of Japanese scolopendromorphs synthesised from the analysis of citizen-sourced data.

Class/Order group			Common name	Number of centipede predation							
				Sm	Sj	Smo	Sa	Ss	Ssp	Os	Scp
VERTEBRATE											
	Amphibia										
		Anura	Frogs/toads	11	1						
		Urodela	Salamanders	1							
	Aves										
		Passeriformes	Swallows	1							
	Mammalia										
		Rodentia	Mice/rats	1							
	Reptilia										
		Squamata	Lizards	28	1						
			Snakes	11							
INVERTEBRATE											
	Arachnida										
		Araneae	Spiders	10	3			1			
		Thelyphonida	Vinegaroons	5							
	Chilopoda										
		Scolopendromorpha	Giant centipedes	45	5						
		Scutigeromorpha	House centipedes	7	1						
	Diplopoda										
		Polydesmida	Flat-backed millipedes		2						1
	Malacostraca										
		Decapoda	Crabs	14			18				
		Isopoda	Pill bug/woodlice	9	13					1	
	Insecta										
		Blattodea	Cockroaches	15	6	1					
		Coleoptera	Beetles	36	30	2					
		Dermaptera	Earwigs	5							
		Diptera	Flies		1						1
		Hemiptera	Aphids	1	1						
			Cicadas	89	21						
			Assassin bugs	4	1						
			Planthoppers							1	
			Leafhoppers	1							
			Shield bugs	10	8						
		Hymenoptera	Ants	2	1						
			Bees/wasps/hornets	7	1						
		Lepidoptera	Moths/butterflies	80	30	2			1	1	4
		Mantodea	Mantises	15	1						
		Megaloptera	Dobsonflies	1							
		Neuroptera	Owlflies	1							
		Odonata	Dragonflies	4							
		Orthoptera	Orthopterans	33	4	2			1		
		Phasmatodea	Walkingsticks	3							
		Unidentified Insecta		11	12	1					
	Unidentified Arthropoda			8	3						1
	Annelida										
		Clitellata	Earthworms	18							7
			Leeches	1							
	Gastropoda										
		Pulmonata	Snails	9	2						1
			Slugs	3							
		Systellommatophora	Leatherleaf slugs	1							
PLANT											
		–	(Flowers)	1	1						
		–	(Fruits)	5	2						
		–	(Saps)	39	18						3
OTHERS											
		–	(Human-derived items)	15		1					
		–	(Scavenging)	80	11	2	2	1	1	1	1
		–	(Unidentified)	27	14				1		

**Note:**

Abbreviations: Os, *Otostigmus scaber*; Sa, *Scolopendra alcyona*; Scp, *Scolopocryptops* sp.; Sj, *Scolopendra japonica*; Sm, *Scolopendra mutilans*; Smo, *Scolopendra morsitans*; Ss, *Scolopendra subspinipes*; Ssp, *Scolopendra* sp.

Predation on vertebrates was confirmed in 55 events, involving a total of 20 species from 13 families (Table 1). Among them, the most common family was geckos, occupying 28.57%, followed by skinks with 12.50%, and grass lizards and snakes with 10.71%.

Predation on invertebrates was confirmed in 641 events, with 87 species from 64 families (Table 1). In insects, which were the most common invertebrate prey items, 30.31% of the observations were predation on true bugs, followed by lepidopterans with 26.11%. The majority (80.29%) of the true bugs identified were cicadas. Many instances of lepidopterans were identified only at the order level because over 70% of them were larvae and were in the process of being consumed by centipedes.

A total of 100 scavenging events (10.85% of all foraging events) were confirmed. In addition, 69 foraging events (7.48%) on plant matter were confirmed, most of which were sap (86.96%), and the rest were fruits and flowers (Table 1). The remaining foraging events included 15 events (1.63%) involving human-derived items (*e.g*., discarded food and insect traps) and 42 events (4.56%) of unidentified items (Table 1).

Of the predation events for which day/night was recorded, 41.28% were day. Five species (*S. mutilans*, *S. japonica*, *S. alcyona*, *S. morsitans*, and *S. subspinipes*) were confirmed to forage both during the day and at night. Both diurnal (*e.g*., skinks) and nocturnal (*e.g*., geckos) species were eaten regardless of the time period of the predation events. Although the foraging environments were diverse, tree trunk (34.40%) was the most common, followed by asphalt/concrete floor (24.37%). A total of 41 foraging events by juveniles were confirmed in *S. mutilans*, *S. japonica* and *S. alcyona*. In this study, individuals showing body colour patterns different from those of adults were categorised as juveniles, regardless of sexual maturity.

Nearly all predation events other than scolopendromorphs (90.38%) were those of house centipedes (Scutigeromorpha). In scutigeromorphs, also the most common prey items were insects, occupying 73.08% of their total foraging events. In insects, 32.89% of the observations were lepidopterans. However, unlike scolopendromorphs, over 90% of lepidopteran prey were adult moths. Only one instance of vertebrate predation by scutigeromorph was confirmed: predation on the Hokou gecko (*Gekko hokouensis*) by *Thereuopoda clunifera*. Although no bias in the time of survey by contributors is expected between scolopendromorphs and scutigeromorphs, daytime foraging by scutigeromorphs was considerably rare (5.32%).

## Discussion

### Feeding ecology of scolopendromorphs

The literature review provided ecologically important data including species names of centipedes and their prey, body size, observation time, and location. Although feeding records of scolopendromorphs have shown a significant increasing trend in recent years, it is highly probable that the available reports are still biased toward conspicuous events, such as predation on vertebrates. This suggests the presence of a publication bias, in which particularly interesting events are preferentially reported. Conversely, in the analysis of citizen-sourced data, although limiting the geographical areas to survey enabled species-level identification for many photographs, the reliability of observation time and geographical information was very variable among the data sources, and quantitative data such as body size were largely unattainable. Nonetheless, the citizen-sourced data complemented the traditional literature survey by revealing a wider range of biotic interactions. Whereas citizen science is also influenced by observer and platform-related biases, it is not constrained by the strong publication bias typical of peer-reviewed reports, and thus provides a different and complementary perspective (Díaz-Calafat et al., 2024). The present study aggregated a large set of published and unpublished records on the dietary range of scolopendromorphs and highlights the effectiveness of combining literature surveys with the analysis of citizen-sourced data in addressing the Eltonian shortfall.

The present results indicate the versatility of scolopendromorphs in foraging, as they consume vertebrates, invertebrates, carcasses, and plant matter. Furthermore, the finding that scolopendromorphs forage in diverse environments and feed on both diurnal and nocturnal animals, during both day and night, indicates that they are highly opportunistic predators. However, although these findings are well supported for *S. mutilans* and *S. japonica*, caution is warranted in generalising them to the order as a whole. Whether the overall foraging versatility reflects opportunistic feeding within each individual species or the accumulation of species-specific prey preferences, remains an open question that requires more systematic, species-specific data.

Nonetheless, a closer look at prey spectra across families provides insight into how this versatility is partitioned within Scolopendromorpha. The interaction networks (Fig. 2) highlight that gastropods were recorded as prey exclusively of Scolopendridae, but not of Cryptopidae, and chilopods were only recorded as prey for Scolopendridae, not for Scolopocryptopidae. These patterns are likely explained by both ecological and observational factors. Scolopendridae are generally larger, enabling them to subdue defended prey such as gastropods and to engage in intraguild predation. In contrast, Scolopocryptopidae and Cryptopidae are smaller, largely subterranean, and may occupy niches with fewer opportunities for such interactions. Additionally, their concealed lifestyle also reduces detectability of predation events, which probably contributes to the underrepresentation of certain prey categories in the records. Whereas the observed family-level differences in prey spectra provide a valuable implication based on evidence, they also illustrate the limits of field observation. Future studies using gut content or DNA-based analyses could help refine and expand our understanding of prey spectra.

The number of scolopendromorph species for which a prey list was compiled using citizen-sourced data is smaller than the total number of scolopendromorph species known from Japan. This likely reflects a bias towards larger, more conspicuous taxa that are more frequently encountered and photographed by non-specialists. Accordingly, the present survey does not capture the full taxonomic and ecological diversity of Japanese centipedes. To address this gap, future research should incorporate targeted field surveys and taxon-specific monitoring.

The consumption of a wide range of prey species is facilitated by the neurotoxins in forcipules of scolopendromorphs. Regarding reptilian prey, scolopendromorphs preyed on various sizes, ranging from smaller to larger than their own body size. Prey snakes tended to be larger than scolopendromorphs, whereas prey lizards were generally smaller. Similar to the present results, many studies have indicated that invertebrate predators using venom have a broader range of prey sizes than nonvenomous solitary predators, allowing the former to capture a wider variety of prey species (McCormick & Polis, 1982; Toledo, Ribeiro & Haddad, 2007; Schalk & Cove, 2018; Suzuki & Mukaimine, 2021). Nonetheless, in the case of eating amphibians and mammals, scolopendromorphs predominantly preyed on only individuals smaller than their own body size. The present results may reflect the fact that possible vertebrate species that can be potentially exploited as prey and are larger than scolopendromorphs are rare except for snakes. *Scolopendra* is one of the largest venomous invertebrates, with certain species exceeding 300 mm in length (Shelley & Kiser, 2000). This size is considered smaller than that of many snake species but larger than that of amphibians, lizards, bats and rodents, which are potential food sources for scolopendromorphs. Indeed, it was reported that *S. heros* in California is larger than all potential lizard prey and 92% of rodent prey (McCormick & Polis, 1982). On the other hand, an alternative hypothesis is that increased body length of snakes may not be an effective defence against centipedes. Prey animals generally improve their ability to escape predation as body size increases (Formanowicz, 1986; Heglund & Taylor, 1988; Irschick & Jayne, 2000; Braña, 2003). However, it has been suggested that in snakes, increasing body length may not effectively reduce vulnerability to a wide range of predators compared to increasing body mass (Schalk & Cove, 2018). Although the present results may lend support to this suggestion that increasing snake body length may not effectively reduce vulnerability to predators, the defensive behaviours of snakes have seldom been examined in relation to invertebrate predators, and it is largely unclear what defensive and escape behaviours snakes exhibit when attacked by centipedes. It should also be noted that the present analysis relied on body length as a proxy for prey size. Because reptiles are generally elongate and slender, whereas many amphibians (anurans) and mammals (*e.g*., bats and rodents) are relatively short-bodied and robust, differences in PPSR among these groups may partly reflect variation in body form rather than true differences in overall body mass or volume.

A feeding strategy that enables the capture and consumption of various organisms is advantageous for expansion into resource-limited environments. In particular, scavenging is efficient for obtaining food. On the other hand, from a human perspective, these feeding strategies make control and extermination of centipedes difficult, suggesting that scolopendromorphs represent a crucial threat as invasive alien species. Indeed, it has been reported that *S. subspinipes*, introduced as a shipping stowaway on Christmas Island, Australia in the 1900s, may have driven the endemic blue-tailed skink (*Cryptoblepharus egeriae*) to extinction (Emery et al., 2021a, 2021b). The problem of invasive alien species has been largely overlooked not only in scolopendromorphs but also in other myriapods (Wittenberg, 2005; Zapparoli & Minelli, 2006; Stoev et al., 2010). These animals are thought to be carried in the gaps between ship cargoes (BBC News, 2005) and in the soil of ornamental plants (Pocock, 1906) and establish populations unnoticed by humans at their point of arrival. As the importance of conserving soil biodiversity has been increasingly recognised (van der Putten et al., 2023), understanding the biological characteristics of hidden invaders, such as centipedes, and developing effective control methods to manage them is becoming ever more crucial.

### Cross-predation

Cross-predation, which refers to one animal becoming either prey or predator to another depending on circumstances, is an important phenomenon for understanding predator–prey dynamics within ecosystems (McCormick & Polis, 1982; Silva-Júnior et al., 2023). Such cross-predation is often exemplified by predation of adult vertebrates on invertebrates that are predators of their own juvenile stages (*e.g*., a dragonfly larva preys on a tadpole, while a frog preys on a dragonfly larva). This is caused by ontogenetic reversals, in which the predator–prey relationship is reversed as the body size of the prey increases over the size of the predator. The present review along with previous studies indicates that scolopendromorphs feed on snakes, lizards, and frogs on the one hand, and these vertebrates are predators of scolopendromorphs on the other hand (Brooks, 1982; Farrell, Smiley-Walters & McColl, 2018; Patharkar, van Passel & Brock, 2022; Xie et al., 2024). However, in the case of centipedes, in which adults often grow larger than adult frogs and lizards, ontogenetic reversals due to the growth of vertebrates, as typically expected like above, may be relatively rare. Rather, reversals in which adult centipedes prey on animals that prey on juvenile centipedes are likely to be more common. In any case, few studies have incorporated such complex and looped interspecific relationships into food webs (McCormick & Polis, 1982).

The present results of the analysis of citizen-sourced data can be compared with those of a recent study (Uno, 2024), which listed predators of scolopendromorphs in Japan, thereby providing a detailed picture of cross-predation among Japanese scolopendromorphs. A comparison of these lists revealed nine species from three classes as both prey and predators of scolopendromorphs (centipedes: *S. mutilans*, *S. japonica*; spiders: *Heteropoda venatoria*, *Typopeltis stimpsonii*; reptiles: *Gekko japonicus*, *Gloydius blomhoffii*, *Goniurosaurus kuroiwae*, *Plestiodon finitimus*, *Takydromus tachydromoides*). At the genus level, an additional five genera from two classes were confirmed (insects: *Gampsocleis* sp., *Tenodera* sp.; amphibians: *Hynobius* sp., *Rana* sp., *Zhangixalus* sp.). Among them, at least *H. venatoria*, *T. tachydromoides*, *P. japonicus*, and *Tenodera* sp., preyed on juvenile centipedes while being preyed upon by adult centipedes. Predation on the enemies of juvenile stages is expected to provide a dual potential benefit. It ensures food resources and reduces the number of future predators of their offspring, which may lead to a long-term impact on population dynamics (McCormick & Polis, 1982).

In recent years, several experimental studies have verified the hypothesis regarding ecological relationships between lizards and centipedes, reporting that both species not only engage in predator–prey interactions, but also compete for acquiring spatial resources such as hiding and foraging places (Charles & Smith, 2009; Emery et al., 2021b; Patharkar, van Passel & Brock, 2022; Xie et al., 2024). On the other hand, interactions of centipedes with snakes have mainly been based on descriptive field reports, with empirical work limited to studies documenting the foraging behaviour of *S. viridis* by pygmy rattlesnakes (*Sistrurus miliarius*) (Farrell, Smiley-Walters & McColl, 2018) and the high resistance of *S. mutilans* to the venom of mamushi (*Gloydius blomhoffii*) (Hamanaka & Mori, 2020). Interactions with spiders are more ambiguous. It has been reported that the experimental removal of spiders in the field led to an increase in the population of scolopendromorphs (Clarke & Grant, 1968). Relationships between spiders and centipedes in the field could represent a fruitful subject for future research. A comprehensive understanding of cross-predation involving centipedes is needed in the future, with ecosystem-level studies anticipated to elucidate the abundance, size thresholds of ontogenetic reversals, and long-term effects of such interactions. Furthermore, multifaceted species-level studies are also needed to explore the contexts in which prey and predators encounter each other, their strategies and tactics, and the effectiveness of their venom.

### Predatory behaviour

The present results show that a relatively large number of scolopendromorphs foraged during the day as well as at night, whereas most foraging events of scutigeromorphs were observed at night. Laboratory studies of circadian rhythms of locomotor activity are very limited in both scutigeromorphs (Tanaka & Watari, 2020) and scolopendromorphs (Cloudsley-Thompson & Crawford, 1970), with no particular differences between them in the frequency of action in the light phase. Daytime foraging increases the risk of desiccation and visual detection by predators. Unlike insects and spiders, centipedes, which lack waxy layers, are vulnerable to desiccation (Lewis, 1981). Among centipedes, scutigeromorphs, which have an exceptionally high tendency to be arboreal and prefer open areas (Dugon, Black & Arthur, 2012), may face a much higher risk of predation during daytime foraging compared to scolopendromorphs. That said, alternative explanations are certainly possible. Future studies that quantitatively assess predation risk will be required to address this issue.

**Figure 4:**
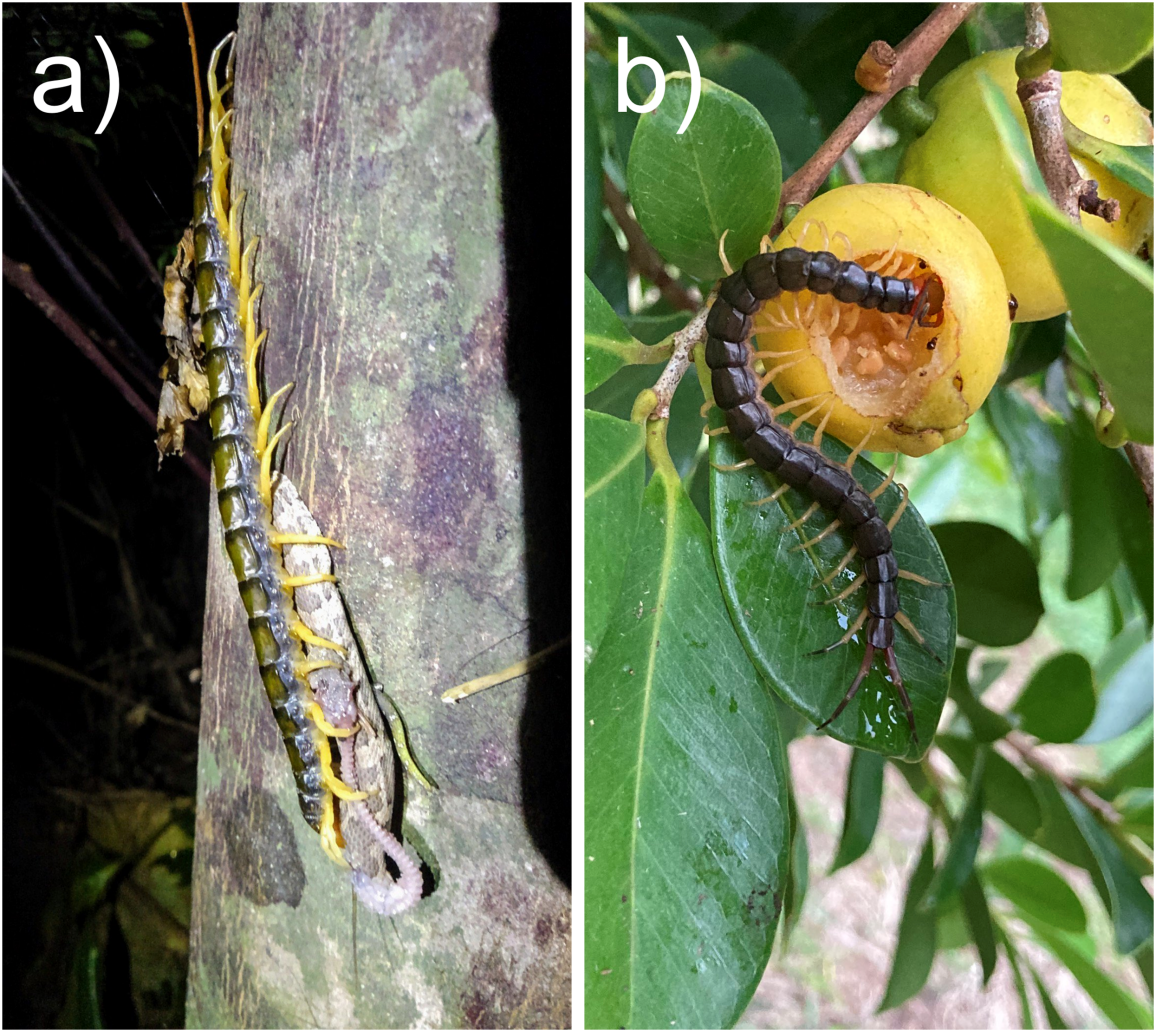
Feeding behaviours of the scolopendromorphs. (A) *Scolopendra mutilans* carrying a pit viper, himehabu (*Ovophis okinavensis*) to a tree trunk in Okinawa Pref. (citation from Oshiro (2018)), and (B) *Scolopendra mutilans* feeding on a guava fruit on a tree on Tanegashima island, Kagoshima Pref. (citation from Saeki (2021)). Images cropped by the author.

The finding that the capture phase was most frequently observed on the ground and the consumption phase in the tree is consistent with the spatial movements of *S. dehaani* with the progression of the predation sequence reported in previous studies (Hodges & Goodyear, 2021). Visual media data also confirmed several photographs and videos of terrestrial and subterranean animals (*e.g*., earthworms, pill bugs and terrestrial snakes) being consumed in trees, on foliage, and on the walls of artificial structures (Fig. 4A). Moving to more exposed surfaces increases predation risk but may help avoid detection by other predatory animals foraging on the ground during the prolonged consumption of large prey (Hodges & Goodyear, 2021; Pwa et al., 2023). For example, footage that recorded *S. mutilans* competing for an earthworm with a ground beetle, which is a powerful, ground-dwelling scavenger, is available (https://x.com/blackpeal7328/status/1548163853145735168).

Cases of fruit and vegetable feeding have been reported based on observations of rearing centipedes (Misra, 1946; Guizze et al., 2016), but these items have not been considered normal food sources in the field (Shinohara & Ehara, 1966). The reliable records of fruit and flower feeding obtained in the present study confirm that at least several Japanese scolopendromorphs feed on plant matter under natural conditions (Fig. 4B). Although fruits are likely consumed for hydration and nutrients such as sugar, the purpose of consuming flowers remains unclear. Similarly, sap is considered to provide hydration and nutritional support. In addition, tree trunks oozing sap attract various insects and might also serve as prime hunting spots. Nonetheless, several sightings of sap-licking centipedes alongside prey animals such as cockroaches and drone beetles have been recorded, suggesting the presence of some factors (*e.g*., hunger) on predatory behaviour decisions in scolopendromorphs.

### Case reports on feeding ecology of scolopendromorphs

A few photographs obtained from the citizen science survey provided valuable insights into the feeding ecology and interspecific relationships of the elusive scolopendromorphs.

**Figure 5:**
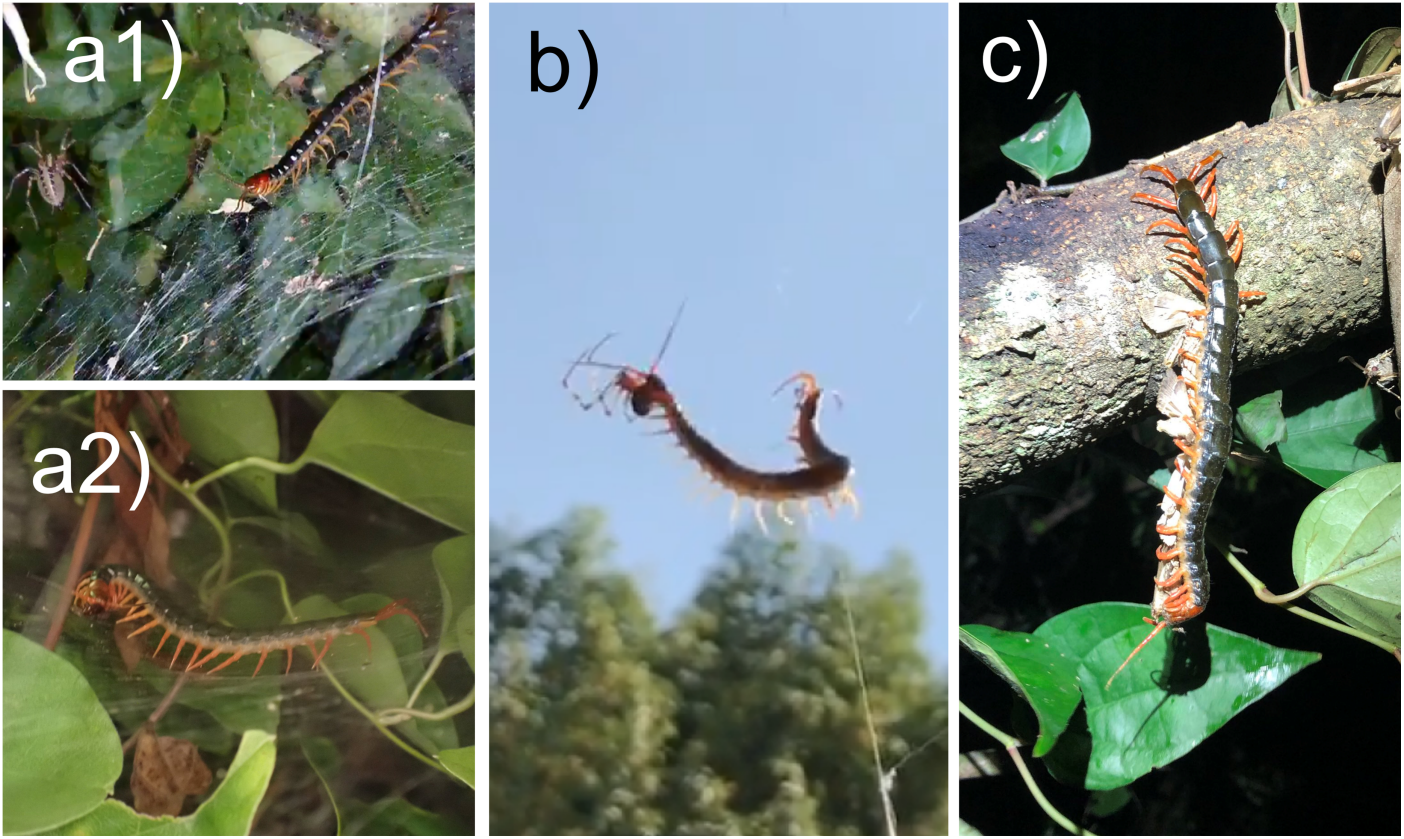
Effective prey-manipulation by scolopendromorphs utilising multiple legs. (A1) scavenging on a spider web of *Agelena* sp. in Osaka Pref. (citation from Yamauchi (2020)), (A2) scavenging on a spider web of Agelenidae in Ishikawa Pref. (citation from Kudo (2021)), (B) attacking a cellar spider on a web suspended in the air (citation from Obayashi (2017)), and (C) capturing and holding multiple moths simultaneously in Fukue-jima island, Nagasaki Pref. (citation from Kojima (2020)). Images (A1) and (B) were generated from videos, and all images were cropped by the author.

Images shown in Fig. 5 provide important information about foraging behaviour unique to chilopods that utilise multiple legs. Scolopendromorphs were occasionally observed foraging on spider webs (*e.g*., Figs. 5A and 5B). Scolopendromorphs invaded spider webs, either intercepting the spider’s prey trapped in the web (scavenging) or attacking the host spider. In the case of scavenging, the host spider remained quietly tucked at the edge of the nest (pers. comm. from the observer). Similar behaviour has been observed in scutigeromorphs (https://x.com/kenji_chinju/status/1584311194244247552). Centipedes have many legs and can still move even when several legs become ensnared in a spider web. In particular, webs made by members of the family Agelenidae (Fig. 5A) may be vulnerable to centipedes because their webs lack sticky droplets and are structured to tangle the legs of prey (Foelix, 2010). Future work could investigate what kind of spider webs centipedes can invade and how easily they forage within spider webs. Simultaneous capture and holding of multiple prey have been reported frequently in scutigeromorphs, but are uncommon in scolopendromorphs (Lewis, 1981). An image in Fig. 5C shows suspended *S. mutilans* holding four adult moths (Noctuidae) using all but six of its hind legs, including the ultimate leg. When the observer (Kojima, Y.) encountered this predation event, the centipede was already in this state, and only fragmentary observation was possible. Hence, it is unclear whether venom had been injected into all the moths, whether the centipede caught them in flight, or how it handled the multiple prey with its many front legs. Currently, little is known about the effective prey handling behaviour using multiple legs, the most basic morphological character of centipedes, other than suppression during conquest (Lewis, 1981).

**Figure 6:**
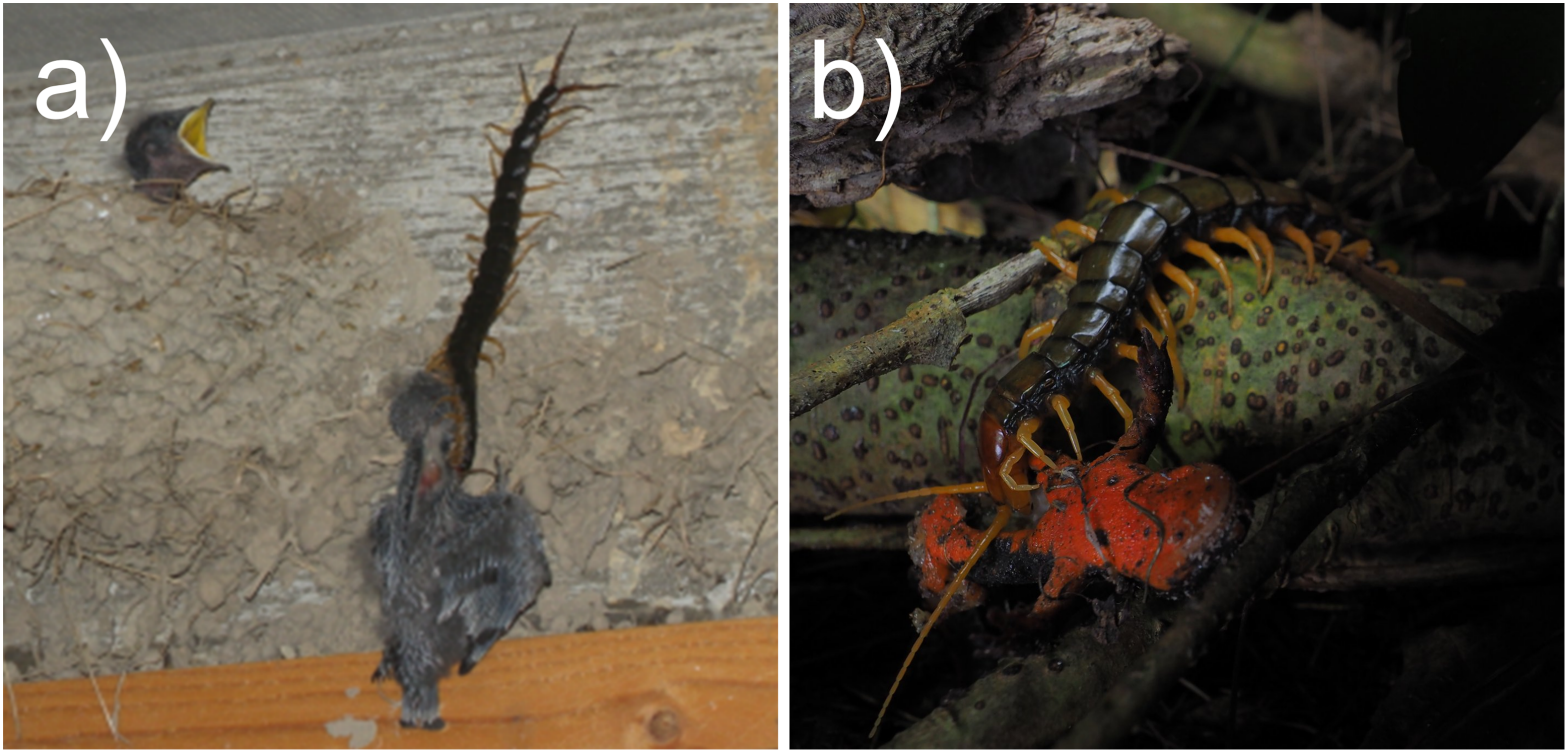
Examples of predation on vertebrates. (A) *Scolopendra mutilans* exploiting a swallow nest and preying on a nestling therein in Shiga Pref. (citation from Terashima (2016)), (B) *Scolopendra mutilans* preying on a poisonous sword-tail newt in Okinawa Pref. (citation from Nasokane (2022)). Images cropped by the author.

An image in Fig. 6A shows the predation on a nestling of the barn swallow (*Hirundo rustica*) by *S. mutilans*, providing the first observation of centipedes climbing a wall and attacking a bird’s nest since a report in 1903 (Cumming, 1903). The identified predator, *S. mutilans*, is not particularly large among the world’s scolopendromorphs, reaching a maximum size of around 150 mm. Therefore, in tropical regions, where larger centipede species are more abundant, birds may be preyed upon more frequently than in Japan.

An image in Fig. 6B shows predation on the sword-tail newt (*Cynops ensicauda*). This newt possesses a strong defensive toxin, and only a few predators are known to eat it (Nakamura & Tominaga, 2021). In addition to the sword-tail newt, other poisonous prey species were confirmed in this review, including millipedes belonging to Julidae, Pachybolidae, and Paradoxosomatidae and uropygids belonging to Thelyphonidae. The effectiveness of their poisons against scolopendromorphs remains unknown. Skin poison of sword-tail newts is known to be ineffective against invertebrates (Calhoun et al., 2017) and has likely evolved in response to vertebrate predation pressure (Jobe, Montaña & Schalk, 2019). Animals that employ specialised defensive tactics, such as the use of poisons, are slower to initiate escape behaviour than those without such defences (Wallace, 1889; Ruxton et al., 2018). As a result, they may be more easily captured by scolopendromorphs and other macroinvertebrates. Documenting predation events on a poisonous animal of which predators are previously unknown provides valuable insights not only into the ecology of predators but also into the evolutionary pressures acting on the prey. The accumulation of opportunistic observations, such as the present ones, will help elucidate the selective forces shaping the evolution of defensive traits and toxin production in well-defended prey, as well as the functions of their specific behaviours.

Predator–prey encounters are rarely observed under natural conditions, and most observations are accidental. The approach used in this study demonstrates the value of citizen science in providing important natural history information to fill knowledge gaps in wildlife research. Individual observations supplement faunal inventories, offering valuable information for research, conservation, and education (Acuña, Isagani & Pitogo, 2021). Although generating new hypotheses and rigorously testing existing hypotheses through the accumulation of numerous sporadic observations demands considerable time and effort, the rapid growth of social networking services and media-sharing platforms in recent years has facilitated the access to vast repositories of wildlife photographs and videos. To deepen our appreciation and knowledge of wildlife, we need to explore efficient methods for handling those media data. More importantly, by conveying the fascination of nature to the public, we should promote field-based exploration involving citizens that fosters the development of scientifically compelling hypotheses.

## Conclusions

By integrating a traditional literature review with an analysis of citizen-sourced data, this study demonstrated that scolopendromorphs exhibit a remarkably broad dietary spectrum, suggesting opportunistic feeding strategies in at least some species. The literature review provided quantitative metrics such as body size and also revealed a clear publication bias towards conspicuous events. In contrast, the analysis of citizen-sourced data compensated for this bias by capturing a broader range of biotic interactions, albeit less quantitative. This finding highlights a potential risk that biological statements based on unverified assumptions and a biased subset of evidence could lead to an underestimation or mischaracterisation of the ecological role, particularly for secretive animals in which direct observations are rare and often accidental. The present study therefore showcases the immense value of citizen science in filling fundamental knowledge gaps for such elusive predators. Ultimately, this integration of traditional literature review and analysis of citizen-sourced data specifically offers a more accurate portrayal of the scolopendromorph ecological niche—thereby contributing to the resolution of the Eltonian shortfall—and broadly advocates a powerful and accessible framework for advancing ecological research in the digital age.

## Supplemental Information

10.7717/peerj.20482/supp-1 Supplemental Information 1 Feeding records of scolopendromorphs under natural conditions based on direct observation.

10.7717/peerj.20482/supp-2 Supplemental Information 2 Feeding records of scolopendromorphs under natural conditions based on indirect observation.

## Additional Information and Declarations

### Data Availability

The following information was supplied regarding data availability:

The raw data are available at Zenodo: Uno, R. (2025). Dataset: Observed prey items of Scolopendromorpha from global literature and Japanese crowdsourced data [Data set]. Zenodo. https://doi.org/10.5281/zenodo.16021806.
