# Supplementary material for: Feeding ecology of scolopendromorphs: integrating a global literature review with Japanese citizen-sourced data
Source: PeerJ. 2026 Jan 5;14:e20482. doi: 10.7717/peerj.20482 (PMC12782032; doi:10.7717/peerj.20482)
Supplement: Supplemental Information 1 [file peerj-14-20482-s001.docx]

| **Supplement 1. Feeding records of scolopendromorphs under natural conditions based on direct observation.** | | | | | | | |
| --- | --- | --- | --- | --- | --- | --- | --- |
| **Higher taxa / Order / Species of prey** | | | **Size of prey** | **Centipede species** | **Size of centipede** | **Predation area (phase)** | **Source** |
| **VERTEBRATE** | | |  |  |  |  |  |
| **Amphibia** | | |  |  |  |  |  |
|  | **Anura** | |  |  |  |  |  |
|  |  | *Dendropsophus elegans* | 26 mm | *Otostigmus tibialis* | 86 mm | top of brick wall (con) | Forti, Fischer & Encarnação 2007* |
|  |  | *Kaloula* cf. *pulchra* | - | *Scolopendra dehaani* | - | ground (con) | Hodges & Goodyear 2021 |
|  |  | *Polypedates leucomystax* | - | *Scolopendra dehaani* | - | liana (con) | Hodges & Goodyear 2021** |
|  |  | *Rhinella marina* | 50 mm | *Ethmostigmus rubripes* | > 120 mm | ground (cap) | Pomeroy et al. 2021** |
|  |  |  | - | *Ethmostigmus rubripes* | - | plant stem (con) | Pikacha & Sirikolo 2012** |
|  |  |  | 95 mm^a)^ | *Scolopendra alternans* | 200 mm | leaf litter (cap) | Carpenter & Gillingham 1984* |
|  |  | *Scinax fuscovarius* | - | *Scolopendra* sp. | - | temporary pond (cap) | Folly et al. 2019*** |
|  |  | Unidentified toad | - | *Scolopendra gigantea* | - | gravel path (con) | Wells-Cole 1898 |
|  |  |  | - | *Scolopendra* sp. | - | - | Daniels unpubul. in Smart, Patel & Pattanayak 2010 |
|  |  | Unidentified frog | - | *Scolopendra* sp. | - | - | Daniels unpubul. in Smart, Patel & Pattanayak 2010 |
|  | **Caudata** | |  |  |  |  |  |
|  |  | *Hynobius setouchi* | 65 mm | *Scolopendra mutilans* | 131 mm^b)^ | under a rotten stump (con) | Jonishi et al. 2023** |
|  |  | *Eurycea quadridigitata* | 75 mm | *Scolopocryptops sexspinosus* | 65 mm | under a log (con) | Palis 2011 |
|  |  | *Speleomantes supramontis* | 50 mm | *Plutonium zwierleini* | 130 mm | cave (tra) | Sanna et al. 2018 |
| **Reptilia** | | |  |  |  |  |  |
|  | **Squamata: Agamidae** | |  |  |  |  |  |
|  |  | *Calotes* sp. | - | Scolopendridae gen. sp. | - | - | Dobroruka 1961 (quoted by Lewis 1966) |
|  | **Squamata: Carphodactylidae** | |  |  |  |  |  |
|  |  | *Underwoodisaurus milii* | 80 mm^a)^ | *Cormocephalus aurantiipes* | 94 mm | under a rock (cap & con) | Kearney & Downes 1998 |
|  | **Squamata: Colubridae** | |  |  |  |  |  |
|  |  | *Calamaria schlegeli* | 150 mm | *Scolopendra subspinipes* | 200 mm | tree trunk (con) | Pwa et al. 2023** |
|  |  |  | - | *Scolopendra subspinipes* | 100 mm | broad leaf (con) | Pwa et al. 2023** |
|  |  | *Coelognathus radiatus* | 610 mm | *Scolopendra* cf. *dehaani* ^c)^ | 290 mm | building floor (cap & con) | Mathipi et al. 2022** |
|  |  | *Dipsas catesbyi* | 245 mm^a)^ | *Scolopendra* sp. | - | leaf litter (con) | von May et al. 2019 |
|  |  | *Leptodeira bakeri* | 626 mm^b)^ | *Scolopendra gigantea* | 180 mm | ground (cap & con) | Buurt & Dilrosun 2017** |
|  |  |  | - | *Scolopendra gigantea* | - | tree trunk (con) | Goessling et al. 2012** |
|  |  | *Lycodon hypsirhinoides* | 80 mm | *Scolopendra dehaani* | 170 mm | front porch of building (con) | Vazifdar, Khalid & D’Costa 2021** |
|  |  | *Lycodon zawi* | 350 mm | *Scolopendra dehaani* | 200 mm | concrete floor (con) | Deb, Sinha & Purkayastha 2023** |
|  |  | *Oligodon taeniolatus* | 360 mm | *Scolopendra hardwickei* | 250 mm | ground (cap) | Smart, Patel & Pattanayak 2010 |
|  |  | *Pituophis catenifer affinis* | 500 mm^a)^ | *Scolopendra heros* | 178 mm | ground (cap) | Babb & Busboom 2013** |
|  |  | *Pseudalsophis biserialis* | 250 mm | *Scolopendra galapagoensis* | 225 mm^d)^ | leaf litter (tra) | Ortiz-Catedral et al. 2021** |
|  |  | *Pseudorabdion longiceps* | - | *Scolopendra subspinipes* | 150 mm | vine and broad leaf (con) | Pwa et al. 2023** |
|  |  | *Rhinocheilus lecontei* | 247 mm | *Scolopendra heros* | 118 mm | road (tra) | Easterla 1975 |
|  |  | *Sibynophis triangularis* | - | *Scolopendra dawydoffi* | - | leaf litter (cap) | Chiacchio et al. 2017** |
|  |  | *Sonora semiannulata* | 218 mm | *Scolopendra heros* | 203 mm | pitfall trap (cap & con) | Johnson, Johnson & Riveroll-Jr 2007** |
|  |  | *Tantilla* sp. | 180 mm^a)^ | *Scolopendra heros* | - | wall crevice (con) | Colacicco & Onishi, 2024** |
|  | **Squamata: Diplodactylidae** | |  |  |  |  |  |
|  |  | *Hesperoedura reticulata* | 78 mm^a)^ | *Scolopendra morsitans* | 84 mm | leaf litter (cap) | Butler 1970 |
|  |  | *Diplodactylus* sp. | 102 mm | *Ethmostigmus* sp. | 89 mm | under a log (con) | Whittell 1883 |
|  | **Squamata: Elapidae** | |  |  |  |  |  |
|  |  | *Bungarus candidus* | 76 mm^a)^ | *Scolopendra subspinipes* | 160 mm | leaf litter (cap) | Radcliffe et al. 2020** |
|  |  | *Calliophis melanurus* | 160 mm | *Scolopendra* sp. | 130 mm | leaf litter (cap), wall crevice (con) | Mirza & Ahmed 2009 |
|  |  | *Hemibungarus mcclungi* | 550 mm | *Scolopendra spinosissima* | 180 mm | ground (cap) | Acuña, Isagani & Pitogo 2021** |
|  |  | *Micrurus obscurus* | >248 mm^a)^ | *Scolopendra* sp. | - | funnel trap (con) | von May et al. 2019 |
|  | **Squamata: Eublepharidae** | |  |  |  |  |  |
|  |  | *Goniurosaurus kuroiwae* | 80 mm | *Scolopendra mutilans* | 115 mm | ground near a stream (con) | Hada 2013** |
|  | **Squamata: Gekkonidae** | |  |  |  |  |  |
|  |  | *Gehyra dubia* | - | *Scolopendra* sp. | - | ground (-) | Nordberg, Edwards & Schwarzkopf 2018** |
|  |  | *Gekko hokouensis* | 56 mm^a)^ | *Scolopendra mutilans* | 120 mm | concrete slope (cap & con) | Okamoto 2019*** |
|  |  | *Gekko hokouensis* | 55 mm^a)^ | *Scolopendra mutilans* | 102 mm | tree trunk (con) | Tanaka 1993 |
|  |  | *Hemidactylus garnotii* | - | *Scolopendra subspinipes* | 152 mm | under a rock (cap) | La Rivers 1948 |
|  |  | *Hemidactylus mercatorius* | - | *Scolopendra subspinipes* | - | building fence (con) | Prötzel, Randriamanana & Glaw 2023** |
|  |  | *Microgecko depressus* | - | *Scolopendra* sp. | - | - | Szczerbak & Golubev 1986 (quoted by Schalk & Cove 2018) |
|  |  | *Pachydactylus* sp. | small | *Scolopendra morsitans* | large | - | Lawrence 1953 |
|  |  | *Phelsuma laticauda* | - | *Scolopendra subspinipes* | - | - | Hawlitschek, Eudeline & Rouillé 2020 (quoted by Prötzel, Randriamanana & Glaw 2023) |
|  |  | Unidentified gecko | - | *Scolopendra* sp. | - | - | Whitaker unpubl. in Smart, Patel & Pattanayak 2010 |
|  | **Squamata: Iguanidae** | |  |  |  |  |  |
|  |  | *Sauromalus ater* | - | *Scolopendra heros* | - | ground (con) | Koleska et al. 2023** |
|  | **Squamata: Lacertidae** | |  |  |  |  |  |
|  |  | *Dalmatolacerta oxycephala* | - | *Scolopendra cingulata* | - | under the rock (con) | Zimić & Jelić 2014** |
|  |  | *Podarcis erhardii* | - | *Scolopendra cingulata* | - | wall crevice (con) | Deimezis-Tsikoutas, Kapsalas & Pafilis, 2020** |
|  |  | *Psammodromus algirus* | 80 mm^a)^ | *Scolopendra oraniensis* | 110 mm | under a rock (-) | Serrano & Farhat, 2023 |
|  | **Squamata: Pareidae** | |  |  |  |  |  |
|  |  | *Pareas carinatus* | - | *Scolopendra dehaani* | - | tree trunk (con) | Siriwut et al. 2016** |
|  | **Squamata: Phrynosomatidae** | |  |  |  |  |  |
|  |  | *Sceloporus olivaceus* | - | *Scolopendra heros* | - | ground (con) | Luna-González, Solís-Rojas & Lazcano 2016** |
|  | **Squamata: Phyllodactylidae** | |  |  |  |  |  |
|  |  | *Gymnodactylus geckoides* | 43 mm | *Scolopendra viridicornis* | 156 mm | falling trap (cap) | Vieira et al. 2021** |
|  | **Squamata: Scincidae** | |  |  |  |  |  |
|  |  | *Cryptoblepharus egeriae* | - | *Scolopendra subspinipes* | - | near building (con) | Emery et al. 2021b** |
|  |  | *Subdoluseps bowringii* | - | *Scolopendra dehaani* | - | rock (cap), tree trunk (con) | Hodges & Goodyear 2021** |
|  | **Squamata: Teiidae** | |  |  |  |  |  |
|  |  | *Ameivula ocellifera* | - | *Scolopendra viridicornis* | - | bucket pitfall trap (con) | Bocchiglieri & Mendonca 2009 |
|  |  |  | 70 mm^a)^ | *Scolopendra* sp. | 143 mm^b)^ | pitfall trap (cap & con) | Moura et al. 2015** |
|  | **Squamata: Xantusiidae** | |  |  |  |  |  |
|  |  | *Xantusia vigilis* | 44 mm | *Scolopendra heros* | 150 mm | - | Sculteure unpubl. in McCormick & Polis 1982 |
|  | **Squamata: Xenodermidae** | |  |  |  |  |  |
|  |  | *Achalinus spinalis* | 238 mm^a)^ | *Scolopendra mutilans* | 91.5 mm | under a rock (con) | Kubo, Fukuyama & Jonishi 2024** |
|  | **Squamata: unidentified** | |  |  |  |  |  |
|  |  | Unidentified snake | - | - | - | building floor (cap) | Okeden 1903** |
| **Aves** | | |  |  |  |  |  |
|  | **Passeriformes** | |  |  |  |  |  |
|  |  | *Icterus icterus* | - | *Scolopendra gigantea* | - | - | Rojas-Suárez unpubul. in Menezes & Marini 2017 |
|  |  | Unidentified sparrow | - | *Scolopendra* sp. | - | bird nest (cap & con) | Cumming 1903 |
|  | **Procellariiformes** | |  |  |  |  |  |
|  |  | *Pterodroma nigripennis* | 44–147 g | *Cormocephalus coynei* | 190 mm ^d)^ | bird nest (cap) | Halpin et al. 2021*** |
|  |  |  | 44–147 g | *Cormocephalus coynei* | 190 mm ^d)^ | bird nest (cap) | Halpin et al. 2021*** |
| **Mammalia** | | |  |  |  |  |  |
|  | **Chiroptera** | |  |  |  |  |  |
|  |  | *Eptesicus furinalis* | 39 mm^e)^ | *Scolopendra viridicornis* | 125 mm^b)^ | building floor (con) | Srbek-Araujo et al. 2012** |
|  |  | *Eptesicus fuscus* | - | *Scolopendra heros* | - | cave ceiling (cap) | Lindley et al. 2017** |
|  |  | *Leptonycteris curasoae* | 54 mm^e)^ | *Scolopendra gigantea* | 210 mm | cave floor (con) | Molinari et al. 2005 |
|  |  | *Molossus molossus* | 36 mm^e)^ | *Scolopendra viridicornis* | 150 mm | building ceiling (cap & con) | Noronha et al. 2015** |
|  |  | *Mormoops megalophylla* | 56 mm^e)^ | *Scolopendra gigantea* | 145 mm | cave ceiling (con) | Molinari et al. 2005** |
|  |  | *Natalus mexicanus* | - | *Scolopendra sumichrasti* | - | cave wall (cap) | Martínez-Coronel, Cupul-Magaña & Nieto-Toscano 2019 |
|  |  |  | - | *Scolopendra sumichrasti* | - | cave ceiling (cap) | Martínez-Coronel, Cupul-Magaña & Nieto-Toscano 2019 |
|  |  |  | - | *Scolopendra sumichrasti* | - | cave wall (con) | Martínez-Coronel, Cupul-Magaña & Nieto-Toscano 2019 |
|  |  | *Pteronotus davyi* | 48 mm^e)^ | *Scolopendra gigantea* | 160 mm | cave ceiling (con) | Molinari et al. 2005 |
|  |  | Unidentified bat | - | *Scolopendra viridicornis* | 140 mm | building roof (con) | Srbek-Araujo et al. 2012 |
|  | **Rodentia** | |  |  |  |  |  |
|  |  | *Aegialomys galapagoensis* | 40 mm^f)^ | *Scolopendra galapagoensis* | 200 mm | rat's nest (cap) | Clark 1979 |
|  |  | *Mus musculus* | 76 mm^b)^ | *Ethmostigmus* sp.^c)^ | 165 mm | under the timber (cap) | Clark, A.H. unpubul. in Shugg 1961** |
| **INVERTEBRATE** | | |  |  |  |  |  |
| **Arachnida** | | |  |  |  |  |  |
|  | **Scorpiones** | |  |  |  |  |  |
|  |  | *Tetratrichobothrius flavicaudis* | 40 mm | *Scolopendra cingulata* | 80 mm | - | Iorio 2006 |
|  |  |  | - | *Scolopendra cingulata* | 90 mm | - | Iorio 2006 |
| **Malacostraca** | | |  |  |  |  |  |
|  | **Decapoda** | |  |  |  |  |  |
|  |  | *Macrobrachium lar* | - | *Scolopendra alcyona* | - | rocks near stream (con) | H. Taira, pers.com. (quoted by Tsukamoto et al. 2021) |
| **Entognatha** | | |  |  |  |  |  |
|  | **Diplura** | |  |  |  |  |  |
|  |  | Unidentified diplura | - | *Cryptops anomalans* | > 70 mm | - | British Myriapod Group 1993 |
| **Insecta** | | |  |  |  |  |  |
|  | **Blattodea** | |  |  |  |  |  |
|  |  | Unidentified cockroaches | - | *Scolopendra abnormis* | - | - | Lewis et al. 2010 |
|  | **Dermaptera** | |  |  |  |  |  |
|  |  | *Forficula auricularia* | - | *Cryptops anomalans* | > 70 mm | - | British Myriapod Group 1993 |
|  | **Hymenoptera** | |  |  |  |  |  |
|  |  | *Tetragonula iridipennis* | - | *Scolopendra hardwickei* ? | 75 mm | beehive box (cap & con) | Karuppasamy, Muthuraman & Jayaraj 2012** |
|  |  | Unidentified ant | - | *Cormocephalus coynei* | 190 mm ^d)^ | - | Halpin et al. 2021 |
|  | **Orthoptera** | |  |  |  |  |  |
|  |  | *Dictyonemobius lateralis* | - | *Cormocephalus coynei* | 190 mm ^d)^ | - | Halpin et al. 2021 |
|  |  | *Dictyonemobius pacificus* | - | *Cormocephalus coynei* | 190 mm ^d)^ | - | Halpin et al. 2021 |
|  |  | *Nesitathra phillipensis* | - | *Cormocephalus coynei* | 190 mm ^d)^ | - | Halpin et al. 2021 |
|  | **unidentified hexapods** | |  |  |  |  |  |
|  |  | Unidentified winged insects | - | *Scolopendra subspinipes* | - | tent peak (cap & con) | Remington 1950 |
| **Chilopoda** | | |  |  |  |  |  |
|  | **Geophilomorpha** | |  |  |  |  |  |
|  |  | *Geophilus flavus* | - | *Cryptops anomalans* | > 70 mm | - | British Myriapod Group 1993 |
|  |  | *Haplophilus subterraneus* | - | *Cryptops anomalans* | > 70 mm | - | British Myriapod Group 1993 |
|  |  | *Henia vesuviana* | - | *Cryptops anomalans* | > 70 mm | - | British Myriapod Group 1993 |
|  | **Scolopendromorpha** | |  |  |  |  |  |
|  |  | *Scolopendra abnormis* | - | *Scolopendra abnormis* | - | - | Cole unpubul. in Tercel et al. 2024 |
|  |  | *Scolopendra subspinipes* | larva | *Scolopendra subspinipes* | mother | under a log (cap) | Sayyed et al. 2022** |
| **Diplopoda** | | |  |  |  |  |  |
|  | **Spirobolida** | |  |  |  |  |  |
|  |  | *Trachelomegalus* sp. | - | *Edentistoma octosulcatum* | - | - | Vahtera & Edgecombe 2014** |
|  | **Polydesmida** | |  |  |  |  |  |
|  |  | *Oxidus gracilis* | - | *Cormocephalus coynei* | 190 mm ^d)^ | - | Halpin et al. 2021 |
|  | **Julida** | |  |  |  |  |  |
|  |  | *Cylindroiulus caeruleocinctus* | - | *Cryptops anomalans* | > 70 mm | - | British Myriapod Group 1993 |
| **Clitellata** | | |  |  |  |  |  |
|  | **Hirudinea** | |  |  |  |  |  |
|  |  | *Orobdella whitmani* | 87 mm | *Scolopendra mutilans* | 80 mm | ground (cap) | Fukuyama & Nakano 2018** |
|  | **Oligochaeta** | |  |  |  |  |  |
|  |  | Unidentified earthworms | - | *Cryptops anomalans* | > 70 mm | - | British Myriapod Group 1993 |
| **Gastropoda** | | |  |  |  |  |  |
|  | **Stylommatophora** | |  |  |  |  |  |
|  |  | *Bekkochlamys* sp. | < 10 mm | *Scolopendra* sp. | 100 mm | tree trunk (con) | Kurozumi & Tanaka, 1986** |
|  | **Systellommatophora** | |  |  |  |  |  |
|  |  | *Laevicaulis alte* | - | *Scolopendra subspinipes* | - | near a streetlamp (con) | Lawrence 1934 |
|  | **unidentified Gastropoda** | |  |  |  |  |  |
|  |  | Unidentified snails | - | *Scolopendra dehaani* | - | tree trunk (con) | Jestrzemski & Schütz 2016 |
| Note: | | - = no data provided, con = consumption, cap = capture, tra = transportation | | | | | |
| ^a)^ | | Values indicate snout–vent length of prey | | | | | |
| ^b)^ | | Measurements taken from photographs using ImageJ | | | | | |
| ^c)^ | | Judged based on photographs, location, and size of the animal | | | | | |
| ^d)^ | | Values indicate summary statistics (e.g., median or mean) reported in the literature. | | | | | |
| ^e)^ | | Values indicate forearm length of prey | | | | | |
| ^f)^ | | Values indicate crown-rump length of prey | | | | | |
| * | | Including illustration of the predatory event | | | | | |
| ** | | Including photograph(s) of the predatory event | | | | | |
| *** | | Including video(s) of the predatory event | | | | | |

**References**

Acuña D, Isagani N, Pitogo KM. 2021. Predation on a McClung’s Philippine false coralsnake, *Hemibungarus mcclungi* (Weigmann 1835), by a giant spiny centipede, *Scolopendra spinosissima* Kraepelin 1903, on Luzon Island, The Philippines. *Reptiles & Amphibians* 28:417–419 DOI: 10.17161/randa.v28i3.15782.

Babb RD, Busboom EC. 2013. *Pituophis catenifer affinis* (Sonoran gophersnake). predation. *Herpetological Review* 44:696.

Bocchiglieri A, Mendonca AF. 2009. *Cnemidophrus ocellifer* (whiptail lizard). predation. *Herpetological Review* 40:438.

British Myriapod Group. 1993. Some notes on *Cryptops anomalans* Newport. *Bulletin of the British Myriapod Group* 9:43.

Butler WH. 1970. From field and study: a record of an invertebrate preying on a vertebrate. *Western Australian Naturalist* 11:146.

Buurt G, Dilrosun H. 2017. Predation by an Amazonian giant centipede (*Scolopendra gigantea*) on a Baker’s cat-eyed snake (*Leptodeira bakeri*). *Reptiles & Amphibians* 24:127 DOI: 10.17161/randa.v24i2.14166

Carpenter C, Gillingham J. 1984. Giant centipede *Scolopendra alternans* attacks marine toad. *Caribbean Journal of Science* 20:71–72.

Chiacchio M, Nadolski BS, Suwanwaree P, Waengsothorn S. 2017. Centipede, *Scolopendra dawydoffi* (Chilopoda: Scolopendridae), predation on an egg-laying snake, *Sibynophis triangularis* (Squamata: Colubridae), in Thailand. *Journal of Insect Behavior* 30:563–566 DOI: 10.1007/s10905-017-9642-0

Clark DB. 1979. A centipede preying on a nestling rice rat (*Oryzomys bauri*). *Journal of Mammalogy* 60:654 DOI: 10.2307/1380119

Colacicco F, Onishi, G. 2024. *Tantilla* sp. (black-headed snake). predation. *Herpetological Review* 55:291–292.

Cumming WD. 1903. The food and poison of a centipede. *The journal of the Bombay Natural History Society* 15:364–365.

Deb A, Sinha D, Purkayastha J. 2023. Predation on Zaw’s wolf snake (*Lycodon zawi*) by a Malaysian cherry red centipede (*Scolopendra dehaani*). *Reptiles & Amphibians* 30:e18468 DOI: 10.17161/randa.v30i1.18468.

Deimezis-Tsikoutas A, Kapsalas G, Pafilis P. 2020. A rare case of saurophagy by *Scolopendra cingulata* (Chilopoda: Scolopendridae) in the central Aegean Archipelago: a role for insularity? *Zoology and Ecology* 30:48–51 DOI: 10.35513/21658005.2020.1.6.

Dobroruka LJ. 1961. *Die Hundertfüßler (Chilopoda)*. Neue Brehm-Bucherei 285. Lutherstadt-Wittenberg: A. Ziemsen Verlag. (in German)

Easterla DA. 1975. Giant desert centipede preys upon snake. *The Southwestern Naturalist* 20:411 DOI: 10.2307/3670046.

Emery J-P, Valentine LE, Hitchen Y, Mitchell N. 2021b. Survival of an extinct in the wild skink from Christmas Island is reduced by an invasive centipede: implications for future reintroductions. *Biological Invasions* 23:581–592 DOI: 10.1007/s10530-020-02386-3.

Folly H, Thaler R, Adams G, Pereira E. 2019. Predation on *Scinax fuscovarius* (Anura, Hylidae) by *Scolopendra* sp. (Chilopoda: Scholopendridae) in the State of Tocantins, Central Brazil. *Revista Latinoamericana de Herpetología* 2:39–43 DOI: 10.22201/fc.25942158e.2019.1.43.

Forti LR, Fischer HZ, Encarnação LC. 2007. Treefrog *Dendropsophus elegans* (Wied-Neuwied, 1824) (Anura: Hylidae) as a meal to *Otostigmus tibialis* Brölemann, 1902 (Chilopoda: Scolopendridae) in the tropical rainforest in southeastern. *Brazilian Journal of Biology* 67:583–584 DOI: 10.1590/S1519-69842007000300028.

Fukuyama I, Nakano T. 2018. The Chinese red-headed centipede *Scolopendra mutilans* (Chilopoda: Scolopendridae) is a predator of the terrestrial macrophagous leech *Orobdella whitmani* (Hirudinida: Orobdellidae). *Edaphologia* 103:33–34 DOI: 10.20695/edaphologia.103.0_33.

Goessling J, Lutterschmidt W, Odum RA, Reinert H. 2012. *Leptodieira bakeri* (Aruban cat-eyed snake). predation. *Herpetological Review* 43:345.

Hada N. 2013. Predation on *Goniurosaurus kuroiwae kuroiwae* by a giant centipede. *Bulletin of the Herpetological Society of Japan* 2013:99–100. (in Japanese)

Halpin LR, Terrington DI, Jones HP, Mott R, Wong WW, Dow DC, Carlile N, Clarke RH. 2021. Arthropod predation of vertebrates structures trophic dynamics in island ecosystems. *The American Naturalist* 198:540–550 DOI: 10.1086/715702.

Hawlitschek O, Eudeline R, Rouillé A. 2020. *Indian Ocean Field Guide: Terrestrial Fauna of the Comoros Archipelago – Guide de Terrain de l’Océan Indien: Faune Terrestre de l’Archipel des Comores*. Hamburg: self-published.

Hodges CW, Goodyear J. 2021. Novel foraging behaviors of *Scolopendra dehaani* (Chilopoda: Scolopendridae) in Nakhon Ratchasima, Thailand. *International Journal of Tropical Insect Science* 41:3257–3262 DOI: 10.1007/s42690-021-00431-9.

Iorio E. 2006. Le scolopendromorphe *Scolopendra cingulata* Latreille, 1829 (Scolopendromorpha, Scolopendridae), un prédateur du scorpion *Euscorpius (Tetratrichobothrius) flavicaudis* (De Geer, 1778) (Scorpiones, Euscorpiidae). *Bulletin d'Arthropoda* 30:60–62. (in French with English abstract)

Jestrzemski D, Schütz S. 2016. Arthropods as predators of herpetofauna in Chu Mom Ray National Park, Vietnam. *Asian Journal of Conservation Biology* 5:3–15.

Johnson JD, Johnson GW, Riveroll-Jr H. 2007. *Sonora semiannulata* (ground snake). predation. *Herpetological Review* 38:93–94.

Jonishi T, Kunihiro Y, van Dung T, Nishikawa K. 2023. *Hynobius setouchi* (Setouchi Salamander). predation. *Herpetological Review* 54:420.

Karuppasamy V, Muthuraman M, Jayaraj R. 2012. Predation of stingless bees (*Trigona iridipennis*: Apidae, Meliponinae) by centipede (*Scolopendra hardwicki*: Chilopoda: Scolopendromorpha). *International Journal of Advanced Life Sciences* 5:156–159.

Kearney M, Downes S. 1998. *Underwoodisaurus milii* (thick-tailed gecko). cross predation. *Herpetological Review* 29:169.

Koleska D, Ertner M, Hammerschmied P, Trávníček O, Holer T, Souder D. 2023. Predation record of a common chuckwalla, *Sauromalus ater* (Iguanidae), by a giant desert centipede, *Scolopendra heros* (Scolopendridae). *Reptiles & Amphibians* 30:e18966 DOI: 10.17161/randa.v30i1.18966.

Kubo G, Fukuyama I, Jonishi T. 2024. *Achalinus spinalis* (Japanese Odd-scaled Snake). predation. *Herpetological Review* 55:268.

Kurozumi T, Tanaka S. 1986. Predation of the helicarionid land snail *Bekkochlamys* sp. by the centipede *Scolopendra* sp. *The biological magazine Okinawa* 24:67–68.

La Rivers I. 1948. Some Hawaiian ecological notes. *Wasmann Collector* 7:85–110.

Lawrence RF. 1953. *The Biology of the Cryptic Fauna of Forests*. Cape Town: August Aimé Balkema.

Lawrence TC. 1934. Notes on the feeding habits of *Scolopendra subspinipes* Leach (Myriopoda). *Proceedings of the Hawaiian Entomological Society* 8:497–498.

Lewis JGE. 1966. The taxonomy and biology of the centipede *Scolopendra amazonica* in the Sudan. *Journal of Zoology* 149:188–203 DOI: 10.1111/j.1469-7998.1966.tb03893.x.

Lewis JGE, Daszak P, Jones C, Cottingham J, Wenman E, Maljković A. 2010. Field observations on three scolopendrid centipedes from Mauritius and Rodrigues (Indian Ocean) (Chilopoda: Scolopendromorpha). *International Journal of Myriapodology* 3:123 DOI: 10.1163/187525410X12578602960425.

Lindley TT, Molinari J, Shelley RM, Steger BN. 2017. A fourth account of centipede (Chilopoda) predation on bats. *Insecta Mundi* 0573:1–4.

Luna-González JMD, Solís-Rojas C, Lazcano D. 2016. *Sceloporus olivaceous* (Texas spiny lizard). predation. *Herpetological Review* 47:469.

Martínez-Coronel M, Cupul-Magaña FG, Nieto-Toscano LF. 2019. Ataques del ciempiés gigante *Scolopendra sumichrasti* Saussure, 1860 (Scolopendromorpha: Scolopendridae) sobre el murciélago Natalus mexicanus Miller, 1902 (Chiroptera: Natalidae) en Chiapas, México. *Acta Zoológica Mexicana* 35:1–5 DOI: 10.21829/azm.2019.3502069. (in Spanish with English abstract)

Mathipi V, Decemson Ht, Biakzuala L, Lalremsanga HT. 2022. *Coelognathus radiatus* (copper-headed trinket snake). predation. *Herpetological Review* 53:506–507.

McCormick S, Polis GA. 1982. Arthropods that prey on vertebrates. *Biological Reviews* 57:29–58 DOI: 10.1111/j.1469-185X.1982.tb00363.x.

Menezes JCT, Marini MÂ. 2017. Predators of bird nests in the Neotropics: a review. *Journal of Field Ornithology* 88:99–114 DOI: 10.1111/jofo.12203.

Mirza ZA, Ahmed JJ. 2009. Note on predation of *Calliophis melanurus* Shaw, 1802 (Serpents: Elapidae) by *Scolopendra* sp. *Hamadryad* 34:166.

Molinari J, Gutiérrez E, De Ascencao A, Nas-Sar J, Arends A, Már-Quez R. 2005. Predation by giant centipedes, *Scolopendra gigantea*, on three species of bats in a Venezuelan cave. *Caribbean Journal of Science* 41:340–346.

Moura LOG, Machado CMS, Conceição BM, Silva AO, Santana AF, Faria RG. 2015. Predation of *Ameivulla ocellifera* (Spix, 1825) (Squamata: Teiidae) by *Scolopendra* sp. (Linneaus, 1758) (Chilopoda: Scholopendridae) in the vegetation of the Caatinga biome, northeastern Brazil. *Herpetology Notes* 8:389–391.

Nordberg EJ, Edwards L, Schwarzkopf L. 2018. Terrestrial invertebrates: an underestimated predator guild for small vertebrate groups. *Food Webs* 15:e00080 DOI: 10.1016/j.fooweb.2018.e00080.

Noronha JC, Battirola LD, Chagas-Jr A, Miranda RM, Carpanedo RS, Rodrigues DJ. 2015. Predation of bat (*Molossus molossus*: Molossidae) by the centipede *Scolopendra viridicornis* (Scolopendridae) in Southern Amazonia. *Acta Amazonica* 45:333–336 DOI: 10.1590/1809-4392201404083.

Okamoto K. 2019. An observation of predation on *Gekko hokouensis* by *Scolopendra mutilans* in the southern part of Kyushu, Japan. *Proceedings of the Kyushu Herpetological Society* 10:7–9. (in Japanese)

Okeden WP. 1903. A centipede eating a snake. *The Journal of the Bombay Natural History Society* 15:135.

Ortiz-Catedral L, Christian E, Chimborazo W, Sevilla C, Rueda D. 2021. A Galapagos centipede *Scolopendra galapagoensis* preys on a floreana racer *Pseudalsophis biserialis*. *Galapagos Research* 70:2–4.

Palis JG. 2011. *Eurycea quadridigitata* (dwarf salamander). centipede predation. *Herpetological Review* 42:405.

Pikacha P, Sirikolo M. 2012. *Bufo marinus* (cane toad). predation. *Herpetological Review* 43:630.

Pomeroy J, Brown G, Webb G, Shine R. 2021. The fauna fights back: invasive cane toads killed by native centipedes in tropical Australia. *Australian Zoologist* 41:738–742 DOI: 10.7882/AZ.2021.002.

Prötzel D, Randriamanana LO, Glaw F. 2023. Invasive centipede (*Scolopendra subspinipes*) preying on a house gecko (*Hemidactylus mercatorius*) in eastern Madagascar. *Spixiana, Zeitschrift für Zoologie* 45:276.

Pwa KH, Yap S, Sind LI, Thong LI, Wong J, Figueroa A. 2023. Predation on two species of reed snakes (Squamata: Colubridae) by the giant forest centipede, *Scolopendra subspinipes* Leach, 1815 (Chilopoda: Scolopendridae), in Singapore. *Herpetology Notes* 16:577–582.

Radcliffe C, Nadolski B, Suwanwaree P, Barnes CH. 2020. *Bungarus candidus* (Malayan krait). predation attempt by centipede. *Herpetological Review* 51:860–861.

Remington CL. 1950. The bite and habits of a giant centipede (*Scolopendra subspinipes*) in the Philippine Islands. *The American Journal of Tropical Medicine and Hygiene* 30:453–455 DOI: 10.4269/ajtmh.1950.s1-30.453.

Sanna L, Bonato L, Marcia P, Zapparoli M. 2018. First record of predation by *Plutonium zwierleini* Cavanna, 1881 (Chilopoda Scolopendromorpha) on *Speleomantes supramontis* (Lanza, Nascetti & Bullini, 1986) (Amphibia Plethodontidae) in Sardinia, Italy. In: *Riassunti Unione Zoologica Italiana 79° Congresso Nazionale Lecce*, 25–28 Settembre 2018. 125.

Sayyed A, Varande A, Solanke Y, Guhagarkar N, Jadhav O, Pawar D, Karjulkar S. 2022. A centipede (*Scolopendra subspinipes*) feeding on its own juveniles. *IOSR Journal of Environmental Science, Toxicology and Food Technology* 16:1–2 DOI: 10.9790/2402-1611010102

Schalk CM, Cove MV. 2018. Squamates as prey: predator diversity patterns and predator-prey size relationships. *Food Webs* 17:e00103 DOI: 10.1016/j.fooweb.2018.e00103.

Serrano FC, Farhat C. 2023. *Psammodromus algirus* (large Psammodromus). predation. *Herpetological Review* 54:666.

Shugg HB. 1961. Predation on the mouse by centipede. *The Western Australian Naturalist* 8:52.

Siriwut W, Edgecombe GD, Sutcharit C, Tongkerd P, Panha S. 2016. A taxonomic review of the centipede genus *Scolopendra* Linnaeus, 1758 (Scolopendromorpha, Scolopendridae) in mainland Southeast Asia, with description of a new species from Laos. *ZooKeys* 590:1–124 DOI: 10.3897/zookeys.590.7950.

Smart U, Patel P, Pattanayak P. 2010. *Scolopendra hardwickei* (Newport, 1844) feeding on *Oligodon taeniolatus* (Jerdon, 1853) in the scrub jungles of Pondicherry, southern India. *Journal of the Bombay Natural History Society* 107:68.

Srbek-Araujo AC, Nogueira M, Lima I, Peracchi A. 2012. Predation by the centipede *Scolopendra viridicornis* (Scolopendromorpha, Scolopendridae) on roof-roosting bats in the Atlantic Forest of southeastern Brazil. *Chiroptera Neotropical* 18:1128–1131.

Szczerbak NN, Golubev ML. 1986. *The Gekkonid Fauna of the U.S.S.R. and Adjacent Countries*. Kiev, Russia: Naukova Dumka Publishing House. (in Russian)

Tanaka S. 1993. Predation of the gecko *Gekko hokouensis* by the centipede *Scolopendra subspinipes*. *Akamata* 9:27. (in Japanese)

Tercel MPTG, Cuff JP, Vaughan IP, Symondson WOC, Goder M, Matadeen S, Tatayah V, Cole NC. 2024. Ecology, natural history, and conservation status of *Scolopendra abnormis*, a threatened centipede endemic to Mauritius. *Endangered Species Research* 54:181–189 DOI: 10.3354/esr01337.

Tsukamoto S, Hiruta SF, Eguchi K, Liao J-R, Shimano S. 2021. A new amphibious species of the genus *Scolopendra* Linnaeus, 1758 (Scolopendromorpha, Scolopendridae) from the Ryukyu Archipelago and Taiwan. *Zootaxa* 4952:465–494 DOI: 10.11646/zootaxa.4952.3.3.

Vahtera V, Edgecombe GD. 2014. First molecular data and the phylogenetic position of the millipede-like centipede *Edentistoma octosulcatum* Tomosvary, 1882 (Chilopoda: Scolopendromorpha: Scolopendridae). PLOS ONE 9:e112461 DOI: 10.1371/journal.pone.0112461.

Vazifdar N, Khalid MA, D’Costa M. 2021. A centipede (*Scolopendra dehaani*) feeding on a juvenile Andaman wolfsnake (*Lycodon hypsirhinoides*) on Havelock Islands, Andaman and Nicobar Islands, India. *Reptiles & Amphibians* 28:341–342. DOI: 10.17161/randa.v28i2.15607.

Vieira W, Gonçalves M, Morais E, Macedo-Jr FV, Vieira K. 2021. Predation of naked-toed gecko, *Gymnodactylus geckoides* Spix, 1825 by giant centipede, *Scolopendra viridicornis* Newport, 1844 in northeastern Brazil (Squamata: Phyllodactylidae). *Herpetological Notes* 14:671–673.

von May R, Biggi E, Cárdenas H, Isabel Diaz M, Alarcon C, Herrera-Alva V, Santa-Cruz R, Tomasinelli F, Westeen EP, Sanchez-Paredes C, Larson J, Title P, Maggie RG, Michael CG, Alison RDR, Rabosky DL. 2019. Ecological interactions between arthropods and small vertebrates in a lowland Amazon rainforest. *Amphibian and Reptile Conservation* 13:65–77.

Wells-Cole B. 1898. Voracious centipede. *Journal of the Bombay Natural History Society* 12:214.

Whittell HR. 1883. On the voracity of a species of heterostoma. *Proceedings of the Linnean Society of New South Wales* 8:33–34 DOI: 10.5962/bhl.part.28639.

Zimić A, Jelić D. 2014. Interspecific illusions: underestimation of the power of the mediterranean banded centipede. *Hyla* 2014:27–29.
